# Description of five new species from southern China, with note on the type species of *Latouchia* Pocock, 1901 (Araneae, Halonoproctidae)

**DOI:** 10.3897/BDJ.12.e137852

**Published:** 2025-01-02

**Authors:** Long Hao, Kun Yu, Feng Zhang

**Affiliations:** 1 Key Laboratory of Zoological Systematics and Application, College of Life Sciences, Hebei University, Baoding, China Key Laboratory of Zoological Systematics and Application, College of Life Sciences, Hebei University Baoding China; 2 Hebei Basic Science Center for Biotic Interaction, Hebei University, Baoding, China Hebei Basic Science Center for Biotic Interaction, Hebei University Baoding China

**Keywords:** Species diversity, embolus, new species, taxonomy, trapdoor spider

## Abstract

**Background:**

The genus *Latouchia* Pocock, 1901 previously included 25 known species and one subspecies from Asia, 12 species and one subspecies were reported in China.

**New information:**

Five new species of *Latouchia* Pocock, 1901 from southern China are described: *L.calcicola* **sp. nov.** (♂♀) from Hainan, *L.jinyun* **sp. nov.** (♂♀) from Chongqing, *L.linmufu* **sp. nov.** (♂♀) from Hunan, *L.wenchuan* **sp. nov.** (♂) from Sichuan and *L.yaoi* **sp. nov.** (♂♀) from south part of Shaanxi. DNA barcodes of the new species described herein are provided. The potential error in the previous illustrations of the alleged male of *L.fossoria* Pocock, 1901 (type species of the genus) is pointed out.

## Introduction

The genus *Latouchia* Pocock, 1901, represents one of the Asian lineages within the family Halonoproctidae Pocock, 1901, forming the subfamily Ummidiinae Ortiz, 2007 alongside the Asian/Australasian genus *Conothele* Thorell, 1878 and the widespread genus *Ummidia* Thorell, 1875 (Godwin et al. 2018). This genus was established, based on *L.fossoria* Pocock, 1901 from Fujian, China (type species) and *L.swinhoei* Pocock, 1901 from the Ryukyu Islands (Pocock 1901), with its type species long mistakenly considered as *L.davidi* (Simon 1886), after Bonnet 1957, until recently corrected (Decae and Caranhac 2020). Concurrently, the re-assessment of several previously proposed morphological differences amongst some described species, such as the stridulatory ridges on chelicerae found only in *L.davidi* and *L.stridulans* Decae, 2019, as well as the unique “demi-saddle” on dorsal side of tibia III recently reported in females of *L.incerta* Decae, Schwendinger & Hongpadharakiree, 2021 and *L.maculosa* Decae, Schwendinger & Hongpadharakiree, 2021, as being more continuously distributed, challenges the monophyly of the genus (Decae and Caranhac 2020; Decae et al. 2021). Relatively cryptic life history traits and selectivity for microhabitats are common characteristics of many trapdoor spiders (Gupta et al. 2015), which may also make specimens of some *Latouchia* species difficult to obtain, thus posing certain obstacles to the exploration of the diversity of this genus.

China stands out as one of the main distribution areas known for *Latouchia*. In the 25 known species and one subspecies within the genus, half (12 species and one subspecies) were reported in China (World Spider Catalog 2024). Nonetheless, recent surveys into mygalomorphs in China suggest that our understanding of *Latouchia* species diversity remains far from complete, with many species awaiting description (unpublished data). This study represents a part of our investigation into *Latouchia* diversity in China, mainly aiming to describe five new *Latouchia* species and provide their DNA barcodes, thereby contributing to future revisions of this poorly-understood group to a certain extent.

## Materials and methods

All specimens preserved in 75%–100% ethanol were examined under a Leica M205A stereomicroscope. The photographs of genitalia were taken by an Olympus BX53 microscope equipped with a Kuy Nice CCD Camera; the photographs of habitus were taken by a SONY Alpha 7R Camera. Photographs of specimens were stacked by the Helicon Focus 8 software and retouched in the Adobe Photoshop CC ©2021 software. Specimens were measured by the measuring tool of Leica LAS V. 4.3 software. All measurements are given in millimetres. Total length of body excludes chelicerae. Spination shown as location (number and arrangement of spines on left leg/right leg), from proximal to distal. The measurements of palps are shown as: total length (femur, patella, tibia, tarsus) (male palpal tarsus measured cymbium only); the measurements of legs are shown as: total length (femur, patella, tibia, metatarsus, tarsus); leg formulae are given as: longest to shortest. Female vulvae were cleared with Pancreatin (BBI Life Sciences). All specimens studied are deposited in the Museum of Hebei University (MHBU), Baoding, China.

Abbreviations used in this study: **A**, apical keel; **AE**, apex of embolus; **ALE**, anterior lateral eye; **AME**, anterior median eye; **BE**, base of embolus; **d**, dorsal; **dK**, dorsal keel; **Dpd**, distal of prodorsal side; **Dpv**, distal of proventral side; **Drd**, distal of retrodorsal side; **Drv**, distal of retroventral side; **K**, keel; **MOA**, median ocular area; **Mp**, middle of prolateral side; **Mpd**, middle of prodorsal side; **Mpv**, middle of proventral side; **Mr**, middle of retrolateral side; **Mrv**, middle of retroventral side; **Mv**, middle of ventral side; **pd**, prodorsal; **pl**, prolateral; **PLE**, posterior lateral eye; **PME**, posterior median eye; **PS**, prolateral superior keel on tip of embolus; **Pv**, proximal of ventral side; **pv**, proventral; **rd**, retrodorsal; **rl**, retrolateral; **RS**, retrolateral superior keel on tip of embolus; **rv**, retroventral; **v**, ventral; **SK**, stalk; **SR**, sperm receptacle; **ST**, subtegulum; **T**, tegulum.

## Taxon treatments

### Latouchia

Pocock, 1901

0C0B4C35-DA04-5CA7-904E-5F299707E0A2

Latouchia fossoria Pocock, 1901Pocock 1901: 210, pl. 21, fig. 2. ; Haupt and Shimojana 2001: 95–110, figs. 1–8. ; Tso et al. 2003: 29, figs. 25–40. ; Decae et al. 2019: 276, figs. 1–52. ; Decae and Caranhac 2020: 563, figs. 1–26. ; Decae et al. 2021: 312, figs. 8–15.

#### Description

**Generic synonyms**

*Kishinouyeus Kishida (1913)* : 22, figs. 8–13; reduced to subgenus of *Latouchia* in Yaginuma (1971): 20; synonym confirmed by Ono and Ogata (2018): 619. Type species: *Kishinouyeustypicus* Kishida, 1913 from Japan.

*Cronebergella* Charitonov (1946): 19, fig. 1; synonym confirmed by Raven (1985): 151. Type species: *Cronebergellakitabensis* Charitonov, 1946 from Uzbekistan.

#### Diagnosis

See Decae et al. (2021): 312.

#### Distribution

China, India, Vietnam, Japan, Thailand, Uzbekistan (?).

#### Notes

*Cronebergella* Charitonov, 1946 was considered as junior synonym of either *Latouchia* or *Sterrhochrotus* Simon, 1892 in two separate publications in 1985 (see Raven (1985): 151 versus Zonstein (1985): 157, respectively). Subsequently, the type species of *Sterrhochrotus* from "Turkestan" has been recombined as *Ummidiaferghanensis* (Kroneberg 1875); hence, *Sterrhochrotus* was considered a junior synonym of *Ummidia* (Decae et al. 2019: 519; World Spider Catalog 2024). However, the type species of *Croneberella* from Uzbekistan is still accepted under *Latouchia* by the World Spider Catalog (2024) as *Latouchiakitabensis* (Charitonov 1946). Other Central Asian Halonoproctidae are instead placed in the genus *Ummidia* Thorell, 1875, including *Ummidiamischi* Zonstein 2014 from Afghanistan and *Ummidiagandjinoi* (Andreeva 1968) from Tajikistan. In this wider context, the taxonomic status of *Croneberella* remains uncertain, this issue needs to be resolved through future re-examination of *L.kitabensis*, especially in the context of all other Central Asian Halonoproctidae.

Schenkel (Schenkel 1963: 14, fig. 1) described *Latouchiavinhiensis* Schenkel, 1963, based on two females preserved in the Muséum national d'Histoire naturelle, Paris (MNHN; not examined), providing textual descriptions and line drawings of the body features, without information on genitalia. The collection information for this species is recorded as “Vinhi, 7. III 1925”, possibly associated with “A. Pichon, Comm. de Douane, 1925” (= Monsieur A. Pichon, Commissioner of customs, 1925) (Schenkel 1963: 8). The specific location referred to by “Vinhi” is puzzling. In the same literature, *Menemerusbonneti* Schenkel, 1963 [now considered a junior synonym of *Menemerusbivittatus* (Dufour 1831)] is another species explicitly described from “Vinhi” and, given that this species is now considered cosmopolitan (World Spider Catalog 2024), it offers little help in tracing back to this location. The original text also mentions several collecting events with nearby dates: “Yunnan-fu, 26. II. 1925”, “Lo Thoei-Tong, 2. III. 1925” and “Tschank-hoa, 10. III. 1925” (Schenkel 1963: 8). “Yunnan-fu” may refer to present-day Kunming City of Yunnan Province, China; perhaps based on potential chronological associations, Wang (2003) listed “Lo Thoei-Tong” as a region under Yunnan Province. This treatment is questionable. First, the collector of “Yunnan-fu, 26. II. 1925” cannot be fully confirmed as A. Pichon, as it is not directly listed under Pichon’s records, but is situated between “Distr. von Yunnan-fu, Plateau v. 1850-2000m Dr Legendre, 1915” and Pichon’s collection records; second, even if all collections in 1925 within Schenkel’s (1963) study were made by Pichon, it cannot be assumed that “Lo Thoei-Tong”, “Vinhi” and “Tschank-hoa” are geographically close to Kunming, Yunnan, because the Yunnan-Vietnam Railway, led by the French, was completed as early as 1910 and, at that time, it took less than three days to travel from Kunming (the starting point) to Haiphong (the endpoint, a port city of northern Vietnam); as a French Indochina customs official, Pichon had both reason and capability to undertake such long-distance travel. The possibility of “Lo Thoei-Tong”, “Vinhi” and “Tschank-hoa” being Vietnamese place names is worth considering, where “Vinhi” and “Tschank-hoa” seem to be phonetically very close to “Vĩnh Hy” and “Khánh Hòa”, with Tỉnh Khánh Hòa including the Nha Trang, an important port city of south-eastern Vietnam and Vĩnh Hy as a famous tourist attraction located about 50 km south of Nha Trang; it is not possible to exclude this possibility based on collection date as, assuming the collector arrived in Haiphong via railway, it would not be difficult to continue to the south-eastern Nha Trang by boat. On the other hand, the spelling of “Vinhi” and “Tschank-hoa” is also like the coastal cities of “Vinh” and “Thanh Hóa” in northern Vietnam. Currently, the distribution of *Latouchiavinhiensis* Schenkel, 1963, is still marked as China on World Spider Catalog (2024) and, based on the above discussion, we remain sceptical about this. However, it is also difficult to determine the specific locations of these place names from the current information and perhaps with future examination of the type specimens, discovery of new specimens or related collectors’ work diaries could provide clues.

### Latouchia calcicola

Hao, Yu & Zhang sp. nov.

5818C014-C5A8-561D-BF90-D1B8B5C03091

4C96961B-959F-43F0-A2D2-883A6BB809C8

#### Materials

- **Type status:** Holotype. **Occurrence:** recordedBy: R. Wen & D. Zhong; sex: male; occurrenceID: 689AB112-C36B-5C5F-977A-82E67476680A; **Taxon:** scientificName: *Latouchiacalcicola* sp. nov.; **Location:** country: China; stateProvince: Hainan; county: Changjiang; locality: Qicha Town, Geming Cave; verbatimElevation: 122 m; verbatimCoordinates: 19.0995°N, 109.0238°E; **Identification:** identifiedBy: L. Hao; **Event:** eventDate: 26 September 2023; **Record Level:** institutionCode: MHBU-ARA-10000046; KYUARA#1977
- **Type status:** Paratype. **Occurrence:** recordedBy: R. Wen & D. Zhong; sex: female; occurrenceID: FBE0DB5F-9524-5198-96B4-604CB23A3656; **Taxon:** scientificName: *Latouchiacalcicola* sp. nov.; **Location:** country: China; stateProvince: Hainan; county: Changjiang; locality: Qicha Town, Geming Cave; verbatimElevation: 122 m; verbatimCoordinates: 19.0995°N, 109.0238°E; **Identification:** identifiedBy: L. Hao; **Event:** eventDate: 26 September 2023; **Record Level:** institutionCode: MHBU-ARA-10000047; KYUARA#1978
- **Type status:** Paratype. **Occurrence:** recordedBy: R. Wen & D. Zhong; sex: female; occurrenceID: 1A17DA56-EA5A-573D-A553-817A0FD3EF62; **Taxon:** scientificName: *Latouchiacalcicola* sp. nov.; **Location:** country: China; stateProvince: Hainan; county: Changjiang; locality: Qicha Town, Geming Cave; verbatimElevation: 122 m; verbatimCoordinates: 19.0995°N, 109.0238°E; **Identification:** identifiedBy: L. Hao; **Event:** eventDate: 26 September 2023; **Record Level:** institutionCode: MHBU-ARA-10000048; KYUARA#2138

#### Description

**Figure 1.**
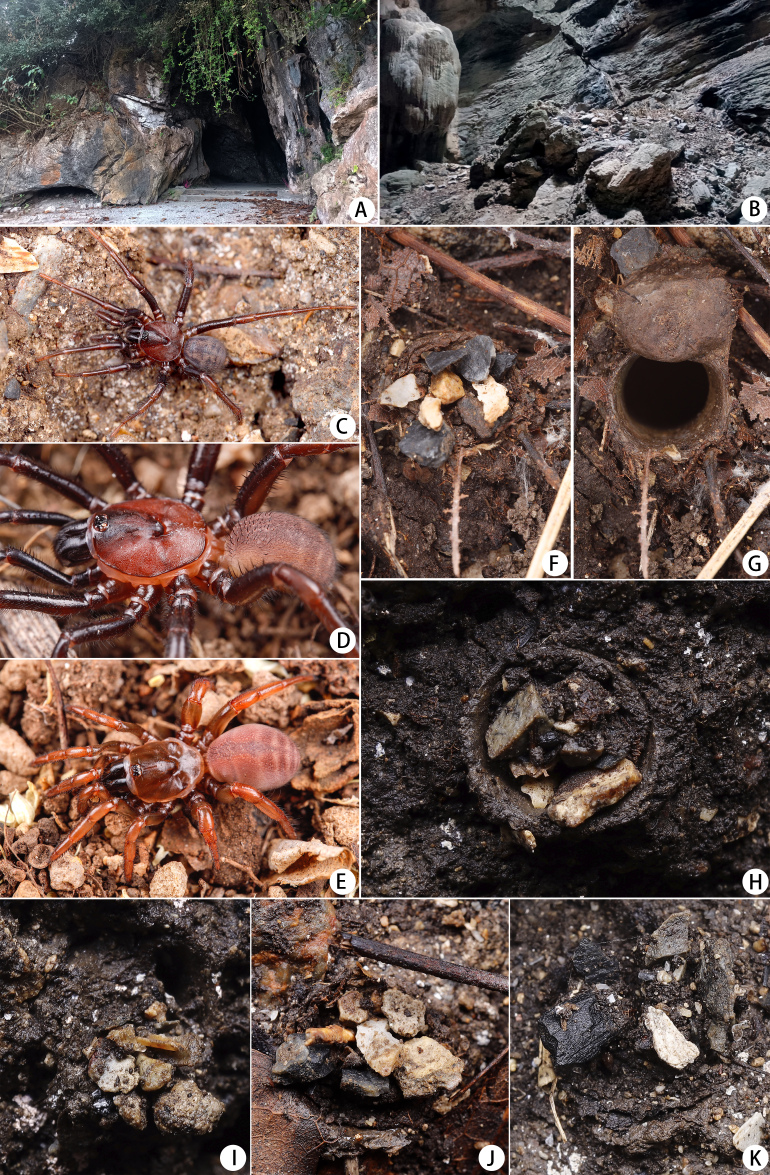
*Latouchiacalcicola* sp. nov. **A, B** Type locality, entrance of Geming Cave (**A**) and the microhabitat (**B**); **C–E** Living spiders, male (**C, D**) and female (**E**); **F–K** Entrances of burrow, with trapdoor closed (**F, H–K**) and opened (**G**), show small stone grains on the trapdoors. Photos: Run Wen.

**Figure 2.**
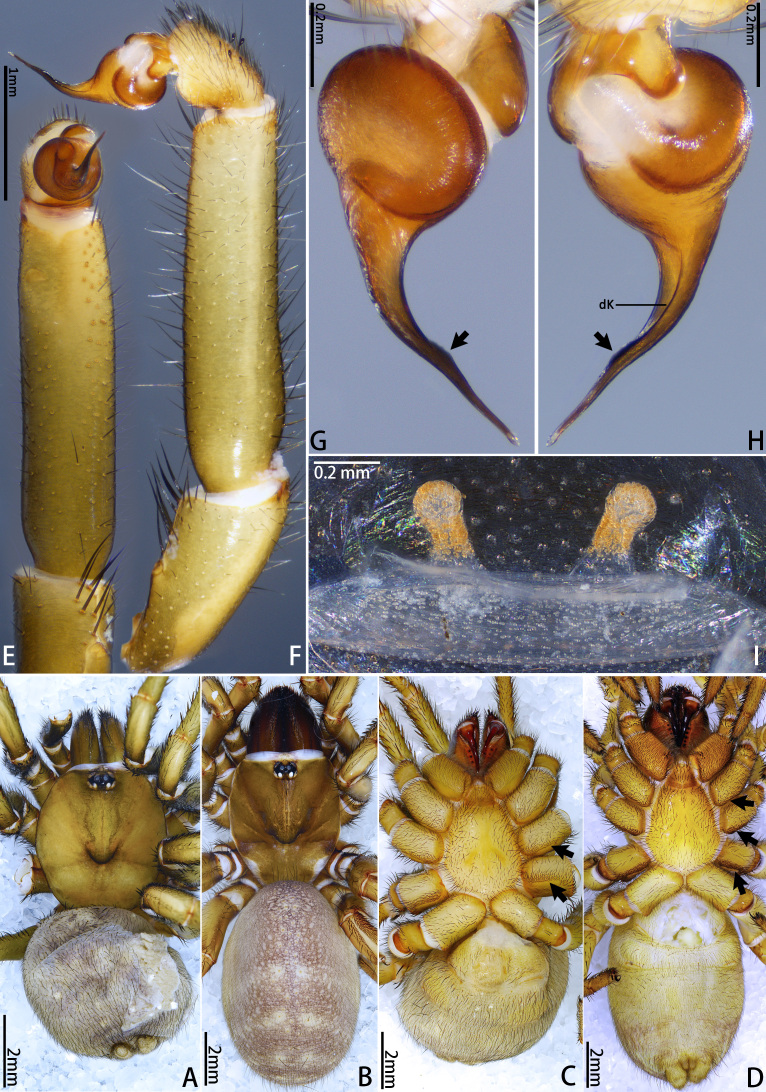
*Latouchiacalcicola* sp. nov. **A**, **C**, **E–H** Holotype male; **B**, **D**, **I.** Paratype female; **A–D** Habitus; **E, F** Palp; **G, H** Details of palpal bulb; **I.** Vulva. In dorsal **(A**, **B**, **I**), retrolateral (**F**, **H**), prolateral (**G**) and ventral (**C–E**) view. Abbreviation: dK, dorsal keel.

**Male** (Holotype, MHBU-ARA-10000046). Colouration in ethanol (Fig. 2A; for colouration of the living male, see Fig. 1C and D). Carapace and chelicerae yellowish-brown, with eye mound, fovea and outer edge of carapace darker; area between eye mound and fovea with two slightly darker longitudinal colour bands. Legs yellowish-brown, with femora gradually transitioning to slightly deeper hue from proximal to distal, darker than rest of legs. Opisthosoma: dorsal side grey, with indistinct dark pattern; ventral side slightly yellower than dorsal side; booklung covers yellower than other ventral areas. Ventral side of whole body generally brighter than dorsal side (Fig. 2C); sigilla slightly darker than rest of sternum; chelicerae ventrally more reddish overall.

Total length 11.23. Carapace 5.31 long, 4.55 wide; opisthosoma 5.92 long, 5.12 wide. Eye group 0.55 long, 0.44 wide anteriorly, 0.71 wide posteriorly; MOA 0.47 long, front width 0.36, back width 0.53. Eye diameters and interdistance: AME 0.15, ALE 0.28, PME 0.22, PLE 0.27, AME–AME 0.15, AME–ALE 0.11, ALE–PLE 0.12, PME–PME 0.32, PME–PLE 0.05. Palpal coxa 1.65 long, 1.04 wide, bearing 12/13 spinules and 4/2 basally thickened bristles on prolateral-proximal corner. Sternum 3.10 long, 2.53 wide. Labium 0.66 long, 0.93 wide, without cuspule or spinule. Chelicerae without stridulatory ridges; rastellum of left and right chelicerae carrying seven and six stout spines, respectively; chelicerae groove with 7/7 and 6/6 teeth of different sizes on promargin and retromargin, respectively.

Leg formula 4123; measurements: I 15.29 (4.94, 2.13, 3.50, 3.10, 1.62), II 13.14 (4.09, 1.70, 3.01, 2.64, 1.70), III 11.25 (2.98, 1.51, 1.82, 3.16, 1.78), IV 15.53 (4.88, 2.09, 3.97, 2.58, 2.01). Spines on femora to metatarsi of legs I–II straight, sword-like (typical); spines on the prolateral patellae of leg I-II typical, both ventrally with few typical; spines on prolateral tibiae of legs I–II typical, but fewer on leg I than II, ventrally typical and expansive on both legs (Fig. 16f). Tarsi I–IV spineless. Spination of leg I, patellae, Dpv (1)/(1), Drv (3)/(1), Mpd (1-1-1)/(1-1-1); tibiae, Dpv (1-1-1)/(1-1-1-2), rv (1-1-2-1-2-2-2)/(1-2-2-2-2); metatarsi, pv (1-1-1-1-1)/(1-1-2-1-1), rv (1-2-1-1-2-1-1)/(2-1-1-1-2-1). Spination of leg II, patellae, Dpv (1)/(2), Drv (1-2)/(1-2), Mpd (1-1-1)/(1-1-1); tibiae, Mp (1-2-1-1)/(1-1-1-1-1), Dpv (1)/(2), Mv (1)/(1), rv (2-2-1-1-1)/(1-1-1-1-1-1-1); metatarsi, pv (1-1-1-1-1-1)/(1-1-1-1), rv (1-1-1-1)/(1-1-1-1). Trichobothria of legs present on proximal one-third part of tibiae I–III, proximal one-fourth part of tibia IV, distal half of metatarsi I–III, distal two-thirds part of metatarsus IV and dorsal side of all tarsi; trichobothria on tibiae I–IV and metatarsi I–IV unmodified; trichobothria on tarsi divided into unmodified and clavate forms, with former irregularly distributed across almost entire dorsal surface and latter only present in proximal half. Count of trichobothria on legs: I, tibia 2/2pd and 2/1rd, metatarsus 6/7, tarsus 13/14 unmodified and 8/8 clavate; II, tibia 5/4pd and 4/4rd, metatarsus 9/9, tarsus 10/12 unmodified and 8/5 clavate; III, tibia 4/3pd and 4/4rd, metatarsus 8/9, tarsus 17/16 unmodified and 3/5 clavate; IV, tibia 4/3pd and 1/1rd, metatarsus 9/5, tarsus 9/5 unmodified and 3/2 clavate. Coxae II–III bear band of spinules on ventro-posterior area. Tarsal claws: paired claws with 3 teeth on tarsi I–III, 3–4 teeth on tarsus IV; unpaired claw bare, without denticle.

**Figure 3.**
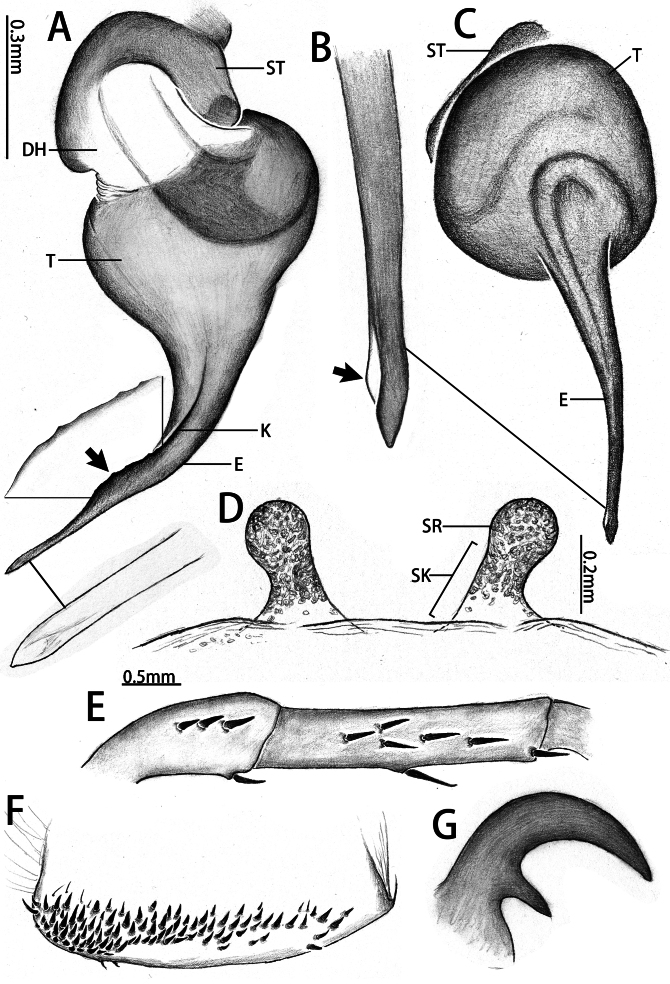
*Latouchiacalcicola* sp. nov. **A–C**, **E–F** Male; **D**, **G** Female. **A, C** Palpal bulb; **B** Distal half of embolus, with arrow indicates the small ridge near the apex of embolus; **D** Vulva; **E** Patella and tibia of left leg II, showing the spination; **F** Band of spinules on coxa III; **G** Claw of left leg I. In retrolateral (**A**), prolateral (**E**, **G**), dorsal **(D**), ventral (**B, C**) and ventro-posterior (**F**) view. Abbreviations: DH, distal haematodocha; E, embolus; K, keel; SR, sperm receptacle; ST–subtegulum; SK–stalk; T–tegulum.

Palp 8.00 long (3.19, 1.48, 2.65, 0.68). Trichobothria on palpal tibia unmodified, present on proximal one-third part, on cymbium divided into unmodified and clavate forms, the former sparsely distributed at distal end of trichobothrial area, while the latter occupies majority of trichobothrial area; count of trichobothria: Tibia 3/3d, cymbium 3/4 unmodified and 8/9 clavate. Tibia slender, moderately uniform in thickness (Fig. 2E and F), with one lyriform organ on ventro-prolateral side of sub-distal part and two adjacent lyriform organs on dorsal side of distal part; tibia lacks spines or spinules. Palpal organ: Tegulum oval (Fig. 2G and H); boundary of tegulum and distal haematodocha visible in retrolateral view of palpal bulb, not reaching base of embolus; embolus long, approximately 1.3 times the width of tegulum, with one distally elevated embolic keel extending from retrolateral side of sub-basal part of embolus to dorsal side of sub-distal part; edge of distal elevation of embolic keel slightly serrulate (Fig. 3A–C); apex of embolus thin, gradually tapering to small and sharp tip.

**Female** (Paratype, MHBU-ARA-10000047). Colouration in ethanol (Fig. 2B; for colouration of living female, see Fig. 1E). Carapace like male, but slightly darker, eye mound and fovea dark; in region near coxa III, outer edge of carapace darkening in colour, appearing burnt red; chelicerae reddish-brown, obviously darker than other body parts. Colour between palp and each leg without significant difference, overall similar in colour to carapace, with coxa III and trochanter III slightly darker. Opisthosoma: Dorsal side reddish-grey, with dense and small irregular pale spots; area around cardiac muscle spot obviously pale. Ventral side of whole body generally brighter than dorsal side (Fig. 2D); colour difference between sigilla and rest of sternum unconspicuous.

Total length 12.95. Carapace 5.13 long, 4.02 wide; opisthosoma 8.93 long, 5.26 wide. Eye group 0.67 long, 0.41 wide anteriorly, 0.66 wide posteriorly; MOA 0.51 long, front width 0.25, back width 0.41. Eye diameters and interdistance: AME 0.13, ALE 0.32, PME 0.24, PLE 0.31, AME–AME 0.12, AME–ALE 0.07, ALE–PLE 0.10, PME– PME 0.25, PME–PLE 0.04. MOA 0.51 long, front width 0.25, back width 0.41. Palpal coxa 2.07 long, 1.17 wide, bearing 20/18 cuspules and 0/1 basally thickened bristle on prolateral-proximal corner. Sternum 3.50 long, 3.17 wide. Labium 1.07 long, 0.90 wide, with two spinules, without cuspule. Chelicerae without stridulatory ridges; rastellum of left and right chelicerae carries 10 and nine stout spines, respectively; chelicerae groove with 5/8 and 7/7 teeth of different sizes on promargin and retromargin, respectively.

Leg formula 4123; measurements: I 9.11 (3.46, 1.77, 2.05, 1.05, 0.78), II 8.04 (3.03, 1.57, 1.58, 1.06, 0.80), III 6.22 (1.73, 0.97, 1.01, 1.38, 1.13), IV 11.38 (3.47, 1.93, 2.57, 1.99, 1.42). Spines of legs I–II primarily distributed on p, pd, r and rv of tibia, as well as p, pv, r and rv of metatarsus and tarsus; most spine tips weakly curved downwards, forming slight hook-shape; some spines on rv longer and not curved at tip. Trichobothria of legs present on proximal one-third part of tibiae I–III, proximal one-fourth part of tibia IV, distal half of metatarsi I–IV and proximal two-thirds of all tarsi; trichobothria on tibiae I–IV and metatarsi I–IV unmodified; trichobothria on tarsi divided into unmodified and clavate forms, with latter only present in proximal half of tarsus. Count of trichobothria on legs: I, tibia 5/5pd and 5/5rd, metatarsus 10/10, tarsus 15/12 unmodified and 11/7 clavate; II, tibia 5/5pd and 5/4rd, metatarsus 11/9, tarsus 11/12 unmodified and 6/7 clavate; III, tibia 4/4pd and 4/4rd, metatarsus 10/12, tarsus 15/11 unmodified and 1/3 clavate; IV, tibia 6/5pd and 5/5rd, metatarsus 8/8, tarsus 12/9 unmodified and ?/3 clavate. Coxae I–III bear band of spinules on ventro-posterior area. Tarsal claws: all paired claws with one tooth (Fig. 3G); unpaired claw bare, without denticle. Palp 8.86 long (3.49, 1.50, 2.08, 1.79), spines on palp distributed on p, pv, r and rv of tibia and tarsus; palpal tarsal claw with one basal tooth.

Vulva (Fig. 2I and Fig. 3D). Two separate sperm receptacles connected to wide and short atrium via slightly tapered stalk; sperm receptacles slightly outwardly inclined. Dense tan glandular pores present on distal half of stalk and throughout entire sperm receptacle. Glandular pores also present on basal half of stalk, atrium, as well as sparsely on the uterus externus, which is nearly transparent in colour.

#### Diagnosis

The male of new species morphologically resembles those of the adjacent mainland (Guandong) species *Latouchiarufa* Zhang & Wang, 2021 in the distinct distal elevation of the dorsal keel of the embolus (dK), forming a lamellar structure; however, it can be distinguished from *L.rufa* as here the dK extends across the proximal two-thirds of the embolus and the length of embolus is approximately 1.3 times the width of bulb (Fig. 2G–H); whereas in *L.rufa*, the dK extends almost to the tip of embolus, the length of embolus is almost equal to the width of bulb (Zhang and Wang (2021): fig. 2B). The female is similar to that of the rather geographically distant Vietnamese species *L.stridulans* Decae, 2019 in overall shape of vulva; however, it can be distinguished by the widest part of the stalk being at the base (Fig. 2I and Fig. 3D), whereas in *L.stridulans*, the widest part of the stalk is in the median part (see Decae et al. (2019): fig. 18). In addition, female also differs from those aspects of *L.stridulans* in the following: the chelicerae lack retrolateral stridulatory ridges and has a band of spinules on the ventro-posterior angle of coxae I to III (also present in the male, Fig. 2C–D and Fig. 3F), whereas in *L.stridulans*, the retrolateral side of the chelicerae has stridulatory ridges and there is no band of spinules at the posterior angle of any coxa. This species also can be distinguished from geographically close *L.wenruni*, *L.yuanjingae* and *L.yejiei* by whether the band of spinules is present at the posterior angle of coxa I to III.

#### Etymology

The specific epithet is Latin, meaning “limestone-dweller”; noun in apposition.

#### Distribution

Known only from the type locality of Hainan, China (Fig. 17).

#### Biology

This newly discovered species constructs burrows in the crevices amongst rocks in the limestone cave of the type locality (Fig. 1A–B). All trapdoors were observed to be covered by small stone grains and some individuals exhibited the presence of small twigs and decomposed leaf fragments on the rim of the burrow entrance (Fig. 1F–K).

#### DNA barcode

AATCATAAAGATATTGGAACTTTATATATAGTGTTAGGGGTATGGTCCGCTATATTGGGTACAGGGATAAGAGTAATAATTCGGATGGAATTAGGACAGGTTGGTAGATTGATTGGAGATGATCATTTGTATAATGTTATTGTTACTGCTCACGCTCTTGTGATGATTTTTTTTATAGTGATGCCTATTATGATTGGGGGATTTGGAAATTGGCTATTGCCTTTGATATTGGGGAGTCCGGATATAGCTTTTCCACGTATGAATAATTTGAGTTTTTGATTATTGCCTCCTTCATTGATGATGTTTTTGATTTCTTCTTTAATTGATACGGGGGTTGGAGCGGGATGGACTATTTATCCTCCTTTGTCTTCTTGTTTGGGGCATAGAGGTGGGGGGATAGATTTTGTTATTTTTTCATTGCATCTAGCAGGGGCTTCTTCAATTATGGGAGCGGTGAATTTTATTTCTACTGTTATGAACATACGTCCTCAGGGAATGAAAATAGAACGGGTTCCTTTGTTTGTGTGATCTGTGTTAATTACGACTATTTTATTATTGTTGTCTTTACCAGTATTGGCGGGGGCAATTACGATATTATTAACTGATCGAAATTTTAATACCTCGTTTTTTGATCCGGCTGGGGGGGGGGATCCGGTGTTGTTTCAACATTTATTTTGATTTTTTGGTCACCC (GenBank accession number: PQ585635).

### Latouchia fossoria

Pocock, 1901

A8C93DA0-469C-5AE3-9672-7D8C56FCF4E8

Latouchia fossoria Pocock 1901: 211, pl. 21, fig. 2; Song et al. 1999: 36 figs. 17 A-B (mislabelled, contra 16Q–R mismatched); Decae and Caranhac 2020: 566, figs. 3, 17–26.

#### Materials

- **Type status:** Holotype. **Occurrence:** recordedBy: John David Digues (de) La Touche; Charles Boughey Rickett; sex: female; occurrenceID: 543E007F-431B-5F5B-A738-4433C800255B; **Location:** country: China; stateProvince: Fujian; county: Nanping; locality: Wuyishan Guadun Village; **Record Level:** institutionCode: British Museum of Natural History (BMNH)

#### Distribution

Known only from the type locality of Fujian, China (Fig. 17).

#### Notes

The *L.fossoria* Pocock, 1901 was originally described, based on a female specimen collected from Kuatun (117.64°E, 27.73°N) in Fujian Province, China. The original illustrations of this species consisted solely of the female's ocular area and sternum (Pocock (1901): pl. 21, figs. 2 and 2a). Song, Zhu & Chen (1999: figs. 16Q–R) provided illustrations of the alleged palp of this species, which has been cited as the male description by the World Spider Catalog (World Spider Catalog 2024). However, the illustrations of the alleged male *L.fossoria* by Song, Zhu & Chen closely match those of the palp of *L.swinhoei* illustrated by Pocock (see Pocock (1901): figs. 3 and 3a), suggesting that the former may be a copy of the latter (Fig. 4). Given the Song, Zhu & Chen cite the right figure numbers from Pocock and as they are not misunderstanding the numbers, we suspect that they have simply reversed the images on the plates, contrary to the intention of the text - the ones said to be (or alleged to be) *L.swinhoei* Pocock, 1901 (figs. 17A-B) are actually the 'missing' images for the female holotype of *L.fossoria*. We believe it is important to highlight this issue, especially considering the significance of *L.fossoria* as the type species for *Latouchia* (Decae and Caranhac 2020). Therefore, the male of *Latouchiafossoria* should currently be considered unknown, requiring further investigation and supplementary descriptions in the future.

### Latouchia jinyun

Hao, Yu & Zhang sp. nov.

EA1D80E7-3DE8-5B31-B8E9-7A0C701743E9

1A5BE02D-B7D4-4F99-8A8E-F126C69B0D37

#### Materials

- **Type status:** Holotype. **Occurrence:** recordedBy: K. Yu & Y. Lin; sex: male; occurrenceID: 60E01C35-E504-57A5-8908-8818E31D1003; **Taxon:** scientificName: *Latouchiajinyun* sp. nov.; **Location:** country: China; stateProvince: Chongqing; county: Beibei; locality: Jinyun Mountain National Nature Reserve; verbatimElevation: 251 m; verbatimCoordinates: 29.8271°N, 106.4307°E; **Identification:** identifiedBy: L. Hao; **Event:** eventDate: 11 January 2016; **Record Level:** institutionCode: MHBU-ARA-10000049; KYUARA#1979
- **Type status:** Paratype. **Occurrence:** recordedBy: K. Yu & Y. Lin; sex: female; occurrenceID: 979CB733-578E-51DA-88C0-8F1E29C24FE1; **Taxon:** scientificName: *Latouchiajinyun* sp. nov.; **Location:** country: China; stateProvince: Chongqing; county: Beibei; locality: Jinyun Mountain National Nature Reserve; verbatimElevation: 251 m; verbatimCoordinates: 29.8271°N, 106.4307°E; **Identification:** identifiedBy: L. Hao; **Event:** eventDate: 11 January 2016; **Record Level:** institutionCode: MHBU-ARA-10000051; KYUARA#2173
- **Type status:** Paratype. **Occurrence:** recordedBy: Z. Li & Z. Yang; sex: female; occurrenceID: 4B02C25E-B0AA-519C-893F-81494D739F16; **Taxon:** scientificName: *Latouchiajinyun* sp. nov.; **Location:** country: China; stateProvince: Chongqing; county: Beibei; locality: Jinyun Mountain National Nature Reserve; verbatimElevation: 376 m; verbatimCoordinates: 29.8382°N, 106.4014°E; **Identification:** identifiedBy: L. Hao; **Event:** eventDate: 12 May 2023; **Record Level:** institutionCode: MHBU-ARA-10000050; KYUARA#1980

#### Description

**Figure 5.**
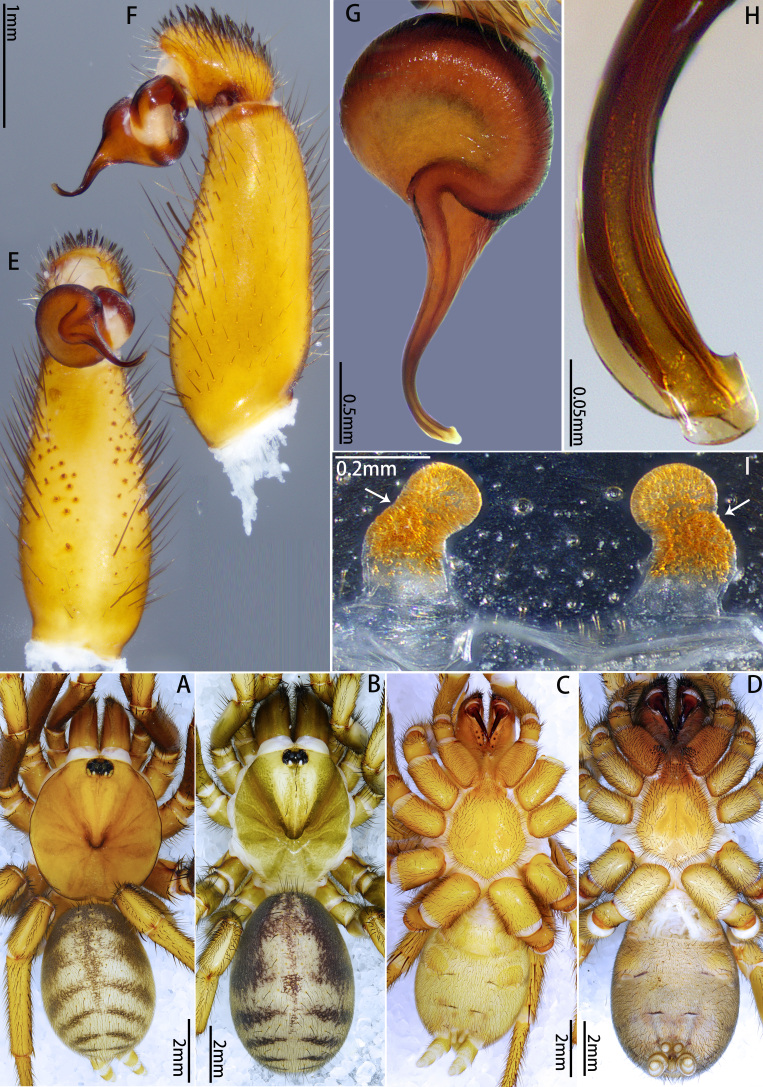
*Latouchiajinyun* sp. nov. **A**, **C**, **E–H** Holotype male; **B**, **D**, **I** Paratype female. **A–D** Habitus; **E, F** Palp; **G** Palpal bulb; **H** Distal half of embolus; **I** Vulva, with arrows indicate stalk tilts approximately 45° towards the body axis. In dorsal (**A**, **B**, **I**), ventral (**C–E**), prolateral (**G**, **H**) and retrolateral (**F**) view.

**Male** (Holotype, MHBU-ARA-10000049). Colouration in ethanol (Fig. 5A). Carapace and chelicerae dark yellow, with eye mound, fovea and outer edge of carapace darker. Legs similar in colour to carapace, with femora gradually transitioning to slightly deeper hue from proximal to distal, darker than rest of legs. Opisthosoma: Dorsal side brownish-grey, with horizontal dark pattern; ventral side yellowish; booklung covers yellower than rest of ventral areas (Fig. 5C). Colour difference between sigilla and rest of sternum unconspicuous.

Total length 11.15. Carapace 5.06 long, 4.78 wide; opisthosoma 6.07 long, 3.98 wide. Eye group 0.61 long, 0.44 wide anteriorly, 0.67 wide posteriorly; MOA 0.42 long, front width 0.25, back width 0.41. Eye diameters and interdistance: AME 0.12, ALE 0.30, PME 0.13, PLE 0.25, AME–AME 0.12, AME–ALE 0.14, ALE–PLE 0.15, PME–PME 0.29, PME–PLE 0.03. Palpal coxa 1.72 long, 0.91 wide, without spinule on prolateral-proximal corner. Sternum 3.03 long, 2.75 wide. Labium 0.56 long, 0.99 wide, without cuspule or spinule. Chelicerae without stridulatory ridges; rastellum of left and right chelicerae carries 10 and nine stout spines, respectively; chelicerae groove with 5/4 and 3/3 teeth of different sizes on promargin and retromargin, respectively.

**Figure 6.**
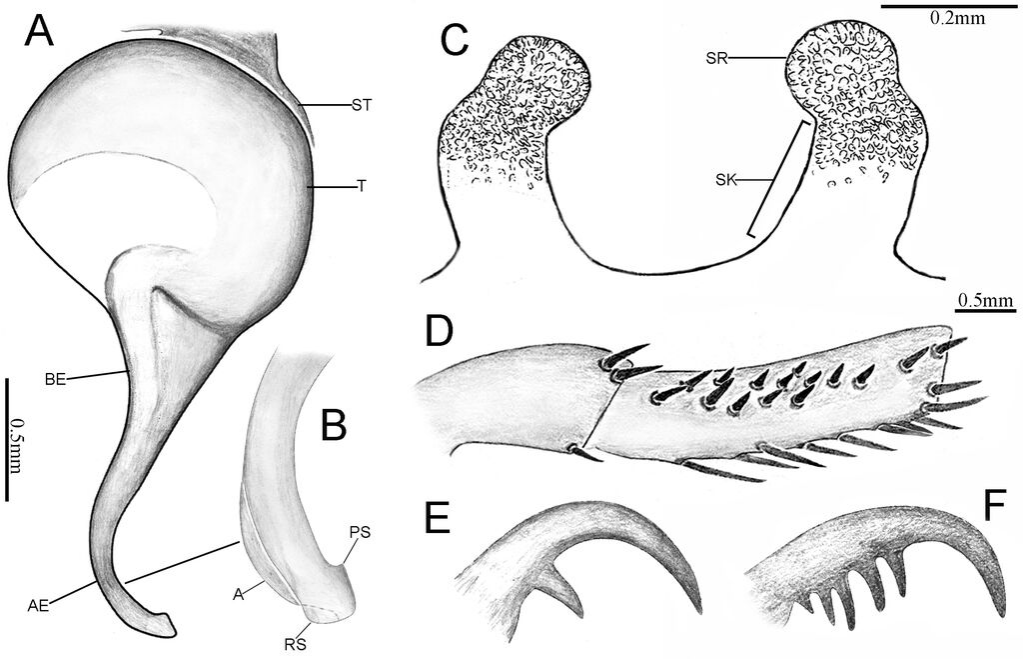
*Latouchiajinyun* sp. nov. **A**, **B**, **D**, **F** Male; **C**, **E.** Female. **A** Palpal bulb; **B** Distal part of embolus; **C** Vulva; **D** Patella and tibia of left leg II, showing the spination; **E, F** Paired claws of left leg I. In prolateral (**A**, **B**, **D**, **E**, **F**) and dorsal (**C**) view. Abbreviations: A, apical keel; AE, apex of embolus; BE, base of embolus; PS, prolateral superior keel on tip of embolus; RS, retrolateral superior keel on tip of embolus; SR–sperm receptacle; ST–subtegulum; SK–stalk; T–tegulum.

Leg formula 4123; measurements: I 12.71 (2.46, 2.11, 3.30, 3.02, 1.82), II 11.36 (2.45, 1.94, 2.61, 2.80, 1.56), III 11.22 (3.34, 1.37, 1.69, 2.68, 2.14), IV 14.74 (4.18, 2.03, 3.46, 3.26, 1.81). Spines on femora to metatarsi of legs I–II straight, sword-like (typical); strong spines on the prolateral patellae of leg II, none on leg I, both ventrally with few typical; spines on prolateral tibiae of legs I–II often short, straight, mostly with hooked tip, much more numerous and stouter on leg II than leg I, ventrally more elongate on both legs, either straight or slightly curved (Fig. 6D and Fig. 16d). Tarsi I–IV spineless. Spination of leg I, patellae, Drv (2)/(1), Dpd (0)/(1); Dpv (1)/(2); tibiae, Dp (1-2)/(1-1-1-2), pv (2-1-2-2)/(1-1-1-1-3-1), rv (1-1-1-1-2)/(1-1-1-2-1); metatarsi, Dpv (1-1)/(1-1), rl (1-1-1)/(1-1-1-1). Spination of leg II, patellae, Dpd (2)/(1), Dpv (1)/(1); tibiae, pl (1-3-1-2-2-2-1-1-1-1)/(1-2-2-3-3-2-2), pv (1-1-1-1-1-3)/(1-1-1-1-1-3), rv (1-1-1-1)/(1-1-1); metatarsi, Mp (1-2)/(1), Dpv (1)/(1), rv (2-1-1-1)/(1-1-1-1). Tibia III unmodified, without demi-saddle shape. Trichobothria of legs present on proximal one-third part of tibiae I–III, proximal one–fourth part of tibia IV, distal half of metatarsi I–III, distal two-thirds part of metatarsus IV and dorsal side of all tarsi; trichobothria on tibiae I–IV and metatarsi I–IV unmodified; trichobothria on tarsi divided into unmodified and clavate forms, with former irregularly distributed across almost entire dorsal surface and latter only present in proximal half. Count of trichobothria on legs: I, tibia 4/5pd and 4/5rd, metatarsus 7/7, tarsus 14/19 unmodified and 5/5 clavate; II, tibia 5/5pd and 6/5rd, metatarsus 8/7, tarsus 16/11 unmodified and 3/3 clavate; III, tibia 5/5pd and 5/4rd, metatarsus 6/7, tarsus 15/14 unmodified and 3/5 clavate; IV, tibia 5/5pd and 6/6rd, metatarsus 7/6, tarsus 10/8 unmodified and 2/1 clavate. Tarsal claws: paired claws with five teeth in different sizes on tarsi I–IV (Fig. 6F); unpaired claw of tarsus I split into two branches; tarsi II–IV unpaired claw bare, without denticle.

Palp 6.48 long (2.41, 1.20, 1.97, 0.90). Trichobothria on palpal tibia unmodified, present on proximal one-third part, on cymbium divided into unmodified and clavate forms, with latter occupying majority of trichobothrial area, while former sparsely distributed at distal end of trichobothrial area; count of trichobothria: tibia 3/3pd, 3/3rd, cymbium 5/4 unmodified and 4/6 clavate. Tibia tubby, slightly diminution from proximal to distal, with one lyriform organ on ventro-prolateral side of sub-distal part; tibia lacks spine or spinule (Fig. 5E–F). Palpal organ: tegulum oval (Fig. 5G); embolus long, approximately 1.5 times the width of tegulum, the distal one-third portion of the embolus is obviously bent, forming the embolus into a hook-shaped structure, with one transparent elevated embolic keel extending from distal one-fourth of embolus to apex; apex of embolus with retrolateral superior keel and prolateral superior keel (Fig. 5H and Fig. 6B).

**Female** (Paratype, MHBU-ARA-10000050). Colouration in ethanol (Fig. 5B). Carapace darker than male, eye mound and fovea dark; area between eye mound and fovea with two slightly lighter longitudinal colour patches; chelicerae similar in colour to carapace. Colour between palp and each leg without significant difference, overall similar in colour to carapace. Opisthosoma: dorsal side as in male; ventral side brighter than dorsal side (Fig. 5D); sigilla slightly darker than rest of sternum; chelicerae ventrally more reddish than male.

Total length 12.97. Carapace 5.53 long, 4.43 wide; opisthosoma 7.43 long, 4.81 wide. Eye group 0.54 long, 0.46 wide anteriorly, 0.60 wide posteriorly; MOA 0.51 long, front width 0.25, back width 0.41. Eye diameters and interdistance: AME 0.15, ALE 0.29, PME 0.15, PLE 0.25, AME–AME 0.12, AME–ALE 0.05, ALE–PLE 0.10, PME– PME 0.29, PME–PLE 0.03, MOA 0.43 long, front width 0.21, back width 0.41. Palpal coxa 1.93 long, 1.23 wide, bearing 44/39 cuspules on prolateral-proximal corner. Sternum 3.09 long, 2.83 wide. Labium 0.76 long, 1.16 wide, with two spinules and one cuspule. Chelicerae without stridulatory ridges; rastellum of left and right chelicerae carrying 11 and nine stout spines, respectively; chelicerae groove with 5/5 and 3/3 teeth of different sizes on promargin and retromargin, respectively.

Leg formula 4123; measurements: I 8.95 (3.25, 1.22, 2.19, 1.27, 1.02), II 8.57 (3.00, 1.57, 1.69, 1.27, 1.04), III 7.54 (2.73, 1.12, 0.93, 1.42, 1.34), IV 15.53 (3.67, 1.58, 2.39, 1.98, 1.74). Spines of legs I–II primarily distributed on p, pd, r and rv of tibia, as well as p, pv, r and rv of metatarsus and tarsus; most spine tips weakly curved downwards, forming slight hook-shape; some spines on rv longer and not curved at tip. Leg III strong; tibia III shortened, without demi-saddle shape. Groups of short spines on apical and prodorsal sides of patella and dorso-lateral side of tibia; metatarsus with spines grouped in dorso-lateral area. Patella with dorso-proximal group of short stiff bristles on leg IV. Trichobothria of legs present on proximal one-third part of tibiae I–IV, distal half of metatarsi I–IV and proximal two-thirds of all tarsi; trichobothria on tibiae I–IV and metatarsi I–IV unmodified; trichobothria on tarsi divided into unmodified and clavate forms, with latter only present in proximal half of tarsus. Count of trichobothria on legs: I, tibia 4/4pd and 5/4rd, metatarsus 7/5, tarsus 10/11 unmodified and 6/6 clavate; II, tibia 5/5pd and 5/5rd, metatarsus 7/7, tarsus 10/10 unmodified and 5/6 clavate; III, tibia 5/5pd and 5/6rd, metatarsus 7/6, tarsus 9/10 unmodified and 2/4 clavate; IV, tibia 7/5pd and 5/6rd, metatarsus 7/7, tarsus 9/7 unmodified and 1/2 clavate. Tarsal claws: all paired claws with one tooth (Fig. 6E); unpaired claw bare, without denticle. Palp 8.29 long (2.55, 1.72, 1.93, 2.09), spines on palp distributed on p, pv, r and rv of tibia and tarsus; palpal tarsal claw with one basal tooth.

Vulva (Fig. 5I and Fig. 6C). Two separate sperm receptacles connected to atrium via slightly outwardly inclined stalk; the distal half of the outer edge of stalk tilts approximately 45° towards the body axis.

#### Diagnosis

The new species can be easily distinguished from the congeners by the following features: in the males, the distal one-third portion of the embolus is obviously bent, forming a hook-shaped structure (Fig. 5G and Fig. 6A); in the females, the distal half of the outer edge of the stalk adjacent to the sperm receptacles tilts 45° towards the body axis in dorsal view (Fig. 5I and Fig. 6C). The arrangement of spines on the prolateral side of male tibia II is similar to that of *L.formosensissmithi*, but differs in that these spines are relatively straight, with no obvious distal curvature and the distal half of the ventral side of tibia II possesses seven to nine spines (Fig. 6D), whereas in *L.formosensissmithi*, the spines on prolateral side of male tibia II exhibits a distinct distal hook-shape and the distal half of the ventral side of tibia II has only one spine (see Tso et al. (2003): fig. 31).

#### Etymology

The specific epithet is derived from the type locality; noun in apposition.

#### Distribution

Known only from the type locality of Chongqing, China (Fig. 17).

#### Biology

The burrows of *L.jinyun* sp. nov. are commonly found on slopes covered with moss along roadsides. The trapdoor is typically composed of a mixture of moss, mud and silk, with few cases of other materials. During a survey conducted in January 2016, many males were found to be overwintering within their burrows, which were covered with trapdoors indistinguishable from those of female.

#### DNA barcode

AAAGATATTGGAACATTGTATTTAGTTTTTGGGGTGTGATCTGCGATATTAGGAACTGGAATAAGAGTAATTATTCGAACTGAGTTGGGGCAGGTGGGGAGAATATTGGGGGATGATCATTTGTATAACGTAATTGTAACTGCTCATGCTCTTGTTATAATCTTTTTTATAGTTATACCTATTATGATTGGGGGATTTGGAAACTGACTACTACCTTTGATATTAGGAGCGCCTGATATAGCATTTCCTCGAATGAATAATTTAAGATTTTGATTGTTACCTCCTTCTTTGTTTATATTGCTTTTGTCTTCATTAGTGGATACTGGGGTTGGAGCAGGTTGGACTATTTATCCGCCTTTATCTTCAGGATTAGGGCATAGAGGTGGAGGAATAGATTTTGCTATTTTTTCTTTACATTTAGCTGGGGCTTCGTCGATTATAGGTTCTATTAATTTTATTTCTACTATTACTAATATGCGTTCTAATGGAATGGATATAGGGCGTGTGCCTTTATTTGTATGGTCTGTGTTAATTACTACTATTTTATTATTATTATCTTTACCCGTTTTGGCTGGAGCTATCACCATATTATTGACAGATCGGAATTTTAATACTTCATTTTTTGACCCTGCGGGGGGTGGGGATCCAATTTTATTTCAACATTTATTTTGATTTTTTGGTCAC (GenBank accession number: PQ585639).

### Latouchia linmufu

Hao, Yu & Zhang sp. nov.

45604915-068A-59F6-9F31-A186C72713D3

04F7CE16-6BDC-4FC1-8DC3-46C8422A7B13

#### Materials

- **Type status:** Holotype. **Occurrence:** recordedBy: J. Lin; sex: male; occurrenceID: BA3628FF-4FC0-55A9-8915-75C521E1F2B2; **Taxon:** scientificName: *Latouchialinmufu* sp. nov.; **Location:** country: China; stateProvince: Hunan; county: Pingjiang; locality: Mufu Mountain National Forest Park; verbatimElevation: 1500 m; verbatimCoordinates: 113.81°E, 28.96°N; **Identification:** identifiedBy: L. Hao; **Event:** eventDate: 19 November 2022; **Record Level:** institutionCode: MHBU-ARA-10000052; KYUARA#1981
- **Type status:** Paratype. **Occurrence:** recordedBy: J. Lin; sex: female; occurrenceID: 38197A48-1DB8-5C7B-BBCF-F5D67BB1D3A6; **Taxon:** scientificName: *Latouchialinmufu* sp. nov.; **Location:** country: China; stateProvince: Hunan; county: Pingjiang; locality: Mufu Mountain National Forest Park; verbatimElevation: 1500 m; verbatimCoordinates: 113.81°E, 28.96°N; **Identification:** identifiedBy: L. Hao; **Event:** eventDate: 19 November 2022; **Record Level:** institutionCode: MHBU-ARA-10000053; KYUARA#1982
- **Type status:** Paratype. **Occurrence:** recordedBy: J. Lin; sex: male; occurrenceID: 812166DB-BEA3-5F1A-BF6A-DEE3114DD382; **Taxon:** scientificName: *Latouchialinmufu* sp. nov.; **Location:** country: China; stateProvince: Hunan; county: Pingjiang; locality: Mufu Mountain National Forest Park; verbatimElevation: 1500 m; verbatimCoordinates: 113.81°E, 28.96°N; **Identification:** identifiedBy: L. Hao; **Event:** eventDate: 19 November 2022; **Record Level:** institutionCode: MHBU-ARA-10000054; KYUARA#1985

#### Description

**Figure 7.**
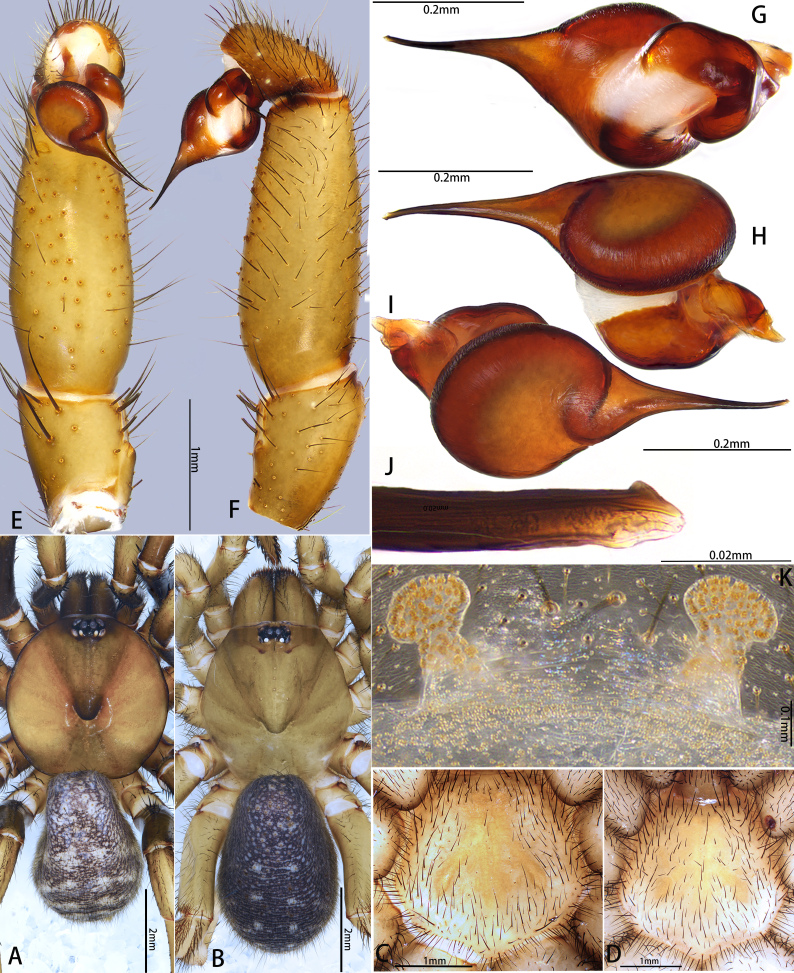
*Latouchialinmufu* sp. nov. **A**, **C**, **E–J.** Holotype male; **B**, **D**, **K** Paratype female. **A, B** Habitus; **C, D.** Sternum; **E, F** Palp; **G–I** Palpal bulb; **J** Apex of embolus; **K** Vulva. In prolateral (**I**, **J**), retrolateral (**F**, **G**), dorsal (**A**, **B**, **K**) and ventral (**C–E**, **H**) view.

**Male** (Holotype, MHBU-ARA-10000052). Colouration in ethanol (Fig. 7A). Carapace and chelicerae yellowish-brown, with eye mound and outer edge of carapace darker; area between eye mound and fovea slightly darker than rest of carapace. Legs yellowish-brown, with femora gradually transitioning to slightly deeper hue from proximal to distal, darker than rest of legs. Opisthosoma: dorsal side black-grey, with indistinct dark pattern; ventral side slightly yellower than dorsal side; booklung covers yellower than other ventral areas. Ventral side of whole body generally brighter than dorsal side; sigilla slightly darker than rest of sternum (Fig. 7C); palpal coxa and labium slightly darker.

Total length 8.97. Carapace 4.68 long, 4.50 wide; opisthosoma 4.25 long, 2.40 wide. Eye group 0.49 long, 0.72 wide anteriorly, 0.74 wide posteriorly; MOA 0.35 long, front width 0.37, back width 0.49. Eye diameters and interdistance: AME 0.14, ALE 0.24, PME 0.10, PLE 0.24, AME–AME 0.08, AME–ALE 0.05, ALE–PLE 0.08, PME–PME 0.28, PME–PLE 0.04. Palpal coxa 1.52 long, 0.94 wide, bearing 9/9 spinules on prolateral-proximal corner. Sternum 2.46 long, 2.35 wide. Labium 0.70 long, 0.81 wide, with one spinule. Chelicerae without stridulatory ridges; rastellum of left and right chelicerae carrying six and four stout spines, respectively; chelicerae groove with 7/5 and 5/4 teeth of different sizes on promargin and retromargin, respectively.

**Figure 8.**
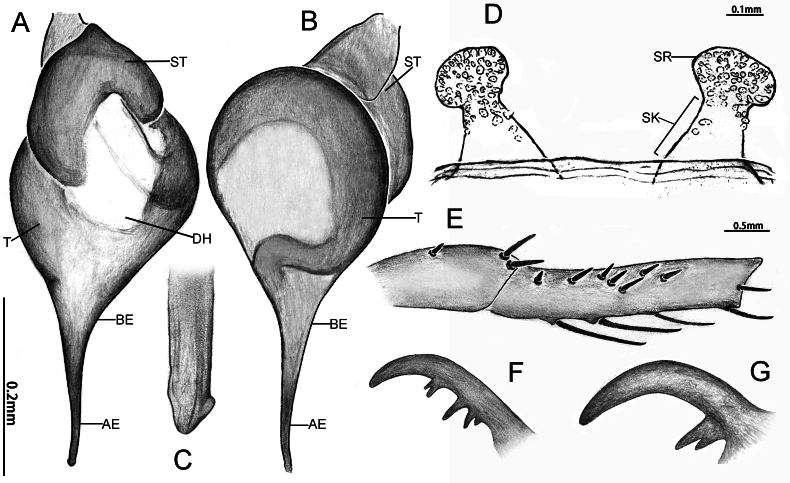
*Latouchialinmufu* sp. nov. **A–C**, **E**, **F** Holotype male; **D**, **G** Paratype female. **A, B** Bulb; **C** Apex of embolus; **D** Vulva; **E** Patella and tibia of left leg II, showing the spination; **F, G** Paired claws of left leg I. In prolateral (**B**, **C**, **E**), retrolateral (**A**, **F**, **G**) and dorsal (**D**) view. Abbreviations: AE, apex of embolus; BE, base of embolus; DH, distal haematodocha; SR–sperm receptacle; ST–subtegulum; SK–stalk; T–tegulum.

Leg formula 4123; measurements: I 14.29 (4.72, 1.70, 3.43, 2.95, 1.49), II 11.86 (3.75, 1.16, 2.81, 2.41, 1.73), III 11.55 (3.41, 1.37, 2.01, 2.91, 1.85), IV 16.99 (5.21, 1.73, 3.71, 3.88, 2.46). Spines on femora to metatarsi of legs I–II straight, sword-like (typical); strong spines on the prolateral patellae of leg I and II, ventrally few typical or absent; spines on prolateral tibiae of legs I–II relatively short, more numerous and stouter on leg II, ventrally more elongate on both legs, three especially elongate spines medially on leg II (Fig. 8E and Fig. 16e); metatarsi I–II spineless and tarsi I–IV spineless. Spination of leg I, patellae, Mpd (1-1-1)/(1-1-2), Dpv (1)/(1), Drv (1)/(1); tibiae, Mp (1-2-1-1)/(2-1), Dpv (1)/(1), v (1-1-1-1-1)/(1-1-1-2). Spination of leg II, patellae, Mpd (1-2)/(1-2); tibiae, Mp (1-1-3-2)/(1-1-1-1-1), Dpv (1)/(1), v (1-1-1-1)/(1-1-1-1). Tibia III unmodified, without demi-saddle shape. Trichobothria of legs present on proximal one-third part of tibiae I–IV, distal half of metatarsi I–III, distal two-thirds part of metatarsus IV and dorsal side of all tarsi; trichobothria on tibiae I–IV and metatarsi I–IV unmodified; trichobothria on tarsi I–III divided into unmodified and clavate forms, with former irregularly distributed across almost entire dorsal surface and latter only present in proximal half, trichobothria on tarsi IV unmodified. Count of trichobothria on legs: I, tibia 3/2pd and 4/2rd, metatarsus 6/5, tarsus 11/11 unmodified and 1/2 clavate; II, tibia 4/4pd and 4/3rd, metatarsus 6/5, tarsus 7/9 unmodified and ?/2 clavate; III, tibia 2/3pd and 3/3rd, metatarsus 4/5, tarsus 5/14 unmodified and ?/2 clavate; IV, tibia 4/5pd and 4/3rd, metatarsus 4/5, tarsus 14/12 unmodified. Tarsal claws: paired claws with five teeth in different sizes on tarsi I–IV (Fig. 8F); unpaired claw bare, without denticle.

Palp 5.39 long (1.76, 1.11, 1.63, 0.89). Trichobothria on palpal tibia unmodified, present on proximal one-third part, on cymbium divided into unmodified and clavate forms, with latter occupying majority of trichobothrial area, while former sparsely distributed at distal end of trichobothrial area; count of trichobothria: tibia 3/2pd, 3/3rd, cymbium 3/4 unmodified and 4/3 clavate. Tibia cylindrical, proximal one-third of tibia widest and slightly narrowing to distal, with one lyriform organ on ventro-prolateral side of sub-distal part (Fig. 7E and F); tibia without spine or spinule. Palpal organ: Tegulum oval (Fig. 7G–J and Fig. 8A–C); the relatively straight and needle-like embolus, gradually narrowing from the proximal to distal end and the absence of obvious keel or ridge on the embolus, the length approximately as the width of tegulum.

**Female** (Paratype, MHBU-ARA-10000053). Colouration in ethanol (Fig. 7B). Carapace like male, but slightly darker, eye mound and fovea dark; area below the eye mound having two bands, burnt red; chelicerae colour as carapace. Colour between palp and each leg without significant difference, overall similar in colour to carapace. Opisthosoma: dorsal side brownish-black, with dense and small irregular pale spots; area around cardiac muscle spot obviously pale. Ventral side of whole body generally brighter than dorsal side; sigilla darker than rest of sternum (Fig. 7D).

Total length 9.89. Carapace 4.88 long, 4.24 wide; opisthosoma 4.99 long, 3.34 wide. Eye group 0.44 long, 0.64 wide anteriorly, 0.68 wide posteriorly; MOA 0.34 long, front width 0.37, back width 0.56. Eye diameters and interdistance: AME 0.14, ALE 0.25, PME 0.12, PLE 0.22, AME–AME 0.09, AME–ALE 0.09, ALE–PLE 0.10, PME– PME 0.31, PME–PLE 0.03. Palpal coxa 1.21 long, 0.77 wide, bearing 13/12 cuspules on prolateral-proximal corner. Sternum 2.59 long, 2.74 wide. Labium 0.72 long, 0.88 wide, without spinule or cuspule. Chelicerae without stridulatory ridges; rastellum of left and right chelicerae carrying 10 and eight stout spines, respectively; chelicerae groove with 5/5 and 5/5 teeth of different sizes on promargin and retromargin, respectively.

Leg formula 4123; measurements: I 8.51 (3.04, 1.92, 1.66, 1.11, 0.78), II 7.75 (2.74, 1.65, 1.312, 1.15, 0.89), III 7.00 (2.30, 1.49, 1.14, 1.22, 0.85), IV 10.50 (3.75, 1.47, 1.99, 1.75, 1.54). Spines of legs I–II primarily distributed on p, pd, r and rv of tibia, as well as p, pv, r and rv of metatarsus and tarsus; most spine tips weakly curved downwards, forming slight hook-shape; some spines on rv longer and not curved at tip. Leg III–IV with tarsus carrying a distal ventral of short stiff bristles. Slender spines on dorso-distal and ventral side of metatarsus III–IV. Tibia III significantly shortened, without demi-saddle shape, groups of short strong spines on dorso-lateral side of tibia and on apical and prodorsal sides of patella III; patella IV with dorso-proximal group of short stiff bristles and few short spines. Trichobothria of legs present on proximal one-third part of tibiae I–IV, distal half of metatarsi I–IV and proximal two-thirds of all tarsi; trichobothria on tibiae I–IV and metatarsi I–IV unmodified; trichobothria on tarsi I–III divided into unmodified and clavate forms, with latter only present in proximal half of tarsus，trichobothria on tarsi IV unmodified. Count of trichobothria on legs: I, tibia 4/4pd and 3/4rd, metatarsus 7/6, tarsus 11/10 unmodified and 3/3 clavate; II, tibia 4/4pd and 4/4rd, metatarsus 7/5, tarsus 10/12 unmodified and 3/1 clavate; III, tibia 3/4pd and 4/4rd, metatarsus 6/7, tarsus 9/12 unmodified and 1/3 clavate; IV, tibia 6/5pd and 5/5rd, metatarsus 5/6, tarsus 8/9 unmodified. Tarsal claws: all paired claws with two teeth on common base (Fig. 8G); unpaired claw bare, without denticle. Palp 5.39 long (1.76, 1.11, 1.63, 0.89), spines on palp distributed on p, pv, r rv of tibia and tarsus; palpal tarsal claw with one basal tooth.

Vulva (Fig. 7K and Fig. 8D). Two separate sperm receptacles connected to atrium via slightly tapered stalk; inner edge at the connection between the stalk and spermathecae is conspicuously concave; sperm receptacles slightly outwardly inclined. Overall mushroom-shaped. Tan glandular pores uniform present on distal half of stalk and entire sperm receptacle. Glandular pores also present on basal half of stalk and atrium, despite being sparser and lighter in colour.

#### Diagnosis

The male of the new species can be distinguished from congeners by the relatively straight and needle-like embolus (Fig. 7G–I), gradually narrowing from the proximal to distal end and the absence of obvious keel or ridge on the embolus, whereas in other species, the embolus may exhibit varying degrees of bending or possess a distinct keel or ridge. The shape of female spermathecae is similar to those of *L.formosensissmithi*, but differs in that the inner edge at the connection between the stalk and spermathecae is conspicuously concave in the dorsal view, whereas in *L.formosensissmithi*, the inner edge of connection between the stalk and spermathecae is smooth, lacking the obvious concavity (see Tso et al. (2003): fig. 40). The female also can be distinguished from the geographically close *L.hunanensis* Xu et al. 2002 by the sperm receptacles slightly outwardly inclined.

#### Etymology

The specific epithet is a combination of the family name of the collector (Lin) and the type locality (Mufu Mountain); noun in apposition.

#### Distribution

Known only from the type locality of Hunan, China (Fig. 17).

#### DNA barcode

GGAAGATTGTTTGGGGATGATCATTTGTATAATGTAATTGTGACTGCACATGCTCTTGTGATGATTTTTTTTATAGTGATGCCTATTATGATTGGCGGTTTTGGCAATTGGTTGTTGCCTATAATGATTGGGGCTCCTGATATGGCATTTCCTCGAATAAATAATTTTAGATTTTGGTTGTTACCTCCTTCTTTGTTTTTGCTTTTGCTGTCTTCTCTAGTGGGTGAGGGGGTTGGAGCAGGGTGAACTATTTATCCTCCCTTGTCTTCGGGGATGGGACATAGAGGAGGGGGTGTGGATTTTGCTATTTTTTCTTTGCATTTAGCGGGGGCGTCCTCAATTATGGGGTCGATTAATTTTATTTCTACTATTATTAATATGCGGGCTAGGGGGATGGATATGGAGAGGGTGCCTTTGTTTGTGTGGTCAGTGTTAATTACTACAGTGTTGCTTTTGTTGTCCTTGCCGGTTCTGGCAGGGGCTATTACGATGTTGTTGACTGATCGTAATTTTAATACTTCGTT (GenBank accession number: PQ585638).

### Latouchia wenchuan

Hao, Yu & Zhang sp. nov.

BAC96581-920F-520D-B609-299209E6AF66

36E66BE9-B60A-4A32-B973-D22773BC5714

#### Materials

- **Type status:** Holotype. **Occurrence:** recordedBy: S. Zheng; sex: male; occurrenceID: F52E6726-346A-534A-9BCF-60F1281E11AC; **Taxon:** scientificName: *Latouchiawenchuan* sp. nov.; **Location:** country: China; stateProvince: Sichuan; county: Wenchuan; locality: near Wenchuan Museum; verbatimElevation: 1377 m; verbatimCoordinates: 31.4706°N, 103.5829°E; **Identification:** identifiedBy: L. Hao; **Event:** eventDate: 27 May 2023; **Record Level:** institutionCode: MHBU-ARA-10000055; KYUARA#1983

#### Description

**Figure 9.**
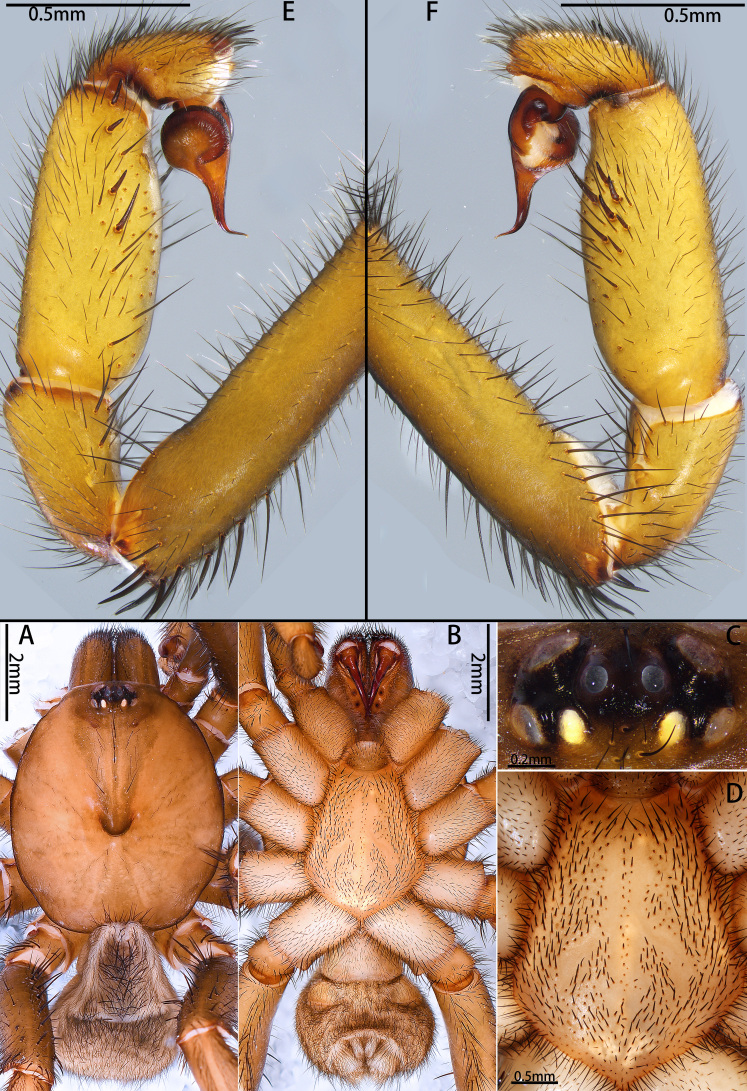
*Latouchiawenchuan* sp. nov. Holotype male. **A, B** Habitus; **C** Ocular area; **D** Sternum; **E, F** Palp. In prolateral (**E**), retrolateral (**F**), dorsal (**A**, **C**) and ventral (**B**, **D**) view.

**Male** (Holotype, MHBU-ARA-10000055). Colouration in ethanol (Fig. 9A). Carapace yellowish-brown, with eye mound (Fig. 9C), fovea, chelicerae and outer edge of carapace darker; area between eye mound and fovea with slightly darker colour subtriangular band. Legs colour as carapace. Opisthosoma: Dorsal side grey; ventral side slightly more yellow than dorsal side (Fig. 9B). Ventral side of whole body generally brighter than dorsal side; sigilla lighter than rest of sternum (Fig. 9D).

Total length 9.22. Carapace 5.03 long, 4.31 wide; opisthosoma 4.17 long, 2.85 wide. Eye group 0.50 long, 0.45 wide anteriorly, 0.68 wide posteriorly; MOA 0.33 long, front width 0.22, back width 0.38. Eye diameters and interdistance: AME 0.11, ALE 0.25, PME 0.15, PLE 0.19, AME–AME 0.14, AME–ALE 0.12, ALE–PLE 0.13, PME–PME 0.30, PME–PLE 0.07. Palpal coxa 1.51 long, 0.81 wide, bearing 18/16 spinules on prolateral-proximal corner. Sternum 3.23 long, 2.46 wide. Labium 0.52 long, 0.87 wide, without cuspule or spinule. Chelicerae without stridulatory ridges; rastellum of left and right chelicerae carrying five and four stout spines, respectively; chelicerae groove with 4/4 and 3/3 teeth of different sizes on promargin and retromargin, respectively.

Leg formula 4123; measurements: I 15.75 (4.84, 2.29, 3.77, 3.21, 1.64), II 12.29 (3.42, 1.94, 2.94, 2.68, 1.31), III 11.60 (3.33, 1.26, 2.05, 2.93, 2.03), IV 16.19 (4.61, 1.49, 3.57, 4.34, 2.18). Spines on femora to metatarsi of legs I–II straight, sword-like (typical); spines on the prolateral patellae of leg I-II typical, ventrally strong on leg I (especially distally), tip hooked, but absent on leg II; spines on prolateral tibiae several typical, less numerous on leg II, ventrally more elongate, two especially elongate adjacent spines proximally on leg II (Fig. 11F and Fig. 16a, b). Short spines on tarsi I–II, spineless III-IV. Spination of right leg I, patellae, Dpv (1), Mrd (1-1-1), rv (1-1-2); tibiae, Drv (2), pv (1-1-2-1-2-2-2-1-2), rl (1-1-2-2-3-1-2); metatarsi, pl (2-2-1-1-1-1-1), rl (2-2-1-1-2-1-2); tarsi, Mp (1-1-1), Mr (1-1-1-1-1). Spination of leg II, patellae, Mpd (1-1-2)/(1-1-2), Drv(1)/(0); tibiae, Mp (1-1-1)/(1-1-1-1), Dpv (2)/(2), rv (2-1-1-1)/(2-1-1-2), Drv (1)/(2); metatarsi, pl(1-1-1-1-1-1-1-1-2)/(1-1-2-1-1-1-3); tarsi, Mp (1-1-1-1-1-1)/(1-1-1-1-1), Mr(1)/(1-1-1). Tibia III unmodified, without demi-saddle shape. Trichobothria of legs present on proximal one-third part of tibiae I–IV, distal half of metatarsi I–III, distal two-thirds part of metatarsus IV and dorsal side of all tarsi; trichobothria on tibiae I–IV and metatarsi I–IV unmodified; trichobothria on tarsi divided into unmodified and clavate forms, with former irregularly distributed across almost entire dorsal surface and latter only present in proximal half. Count of trichobothria on legs: I, tibia 3pd and 2rd, metatarsus 7, tarsus 14 unmodified and 4 clavate; II, tibia 4/5pd and 3/4rd, metatarsus 7/7, tarsus 11/15 unmodified and 3/7 clavate; III, tibia 4/5pd and 4/5rd, metatarsus 7/8, tarsus 13/13 unmodified and 1/3 clavate; IV, tibia 5/5pd and 6/6rd, metatarsus 7/8, tarsus 11/10 unmodified and 1/1 clavate. Tarsal claws: paired claws with six teeth on tarsus I (Fig. 11E), four teeth on tarsi II–III, two teeth on tarsus IV, unpaired claw bare, without denticle.

**Figure 10.**
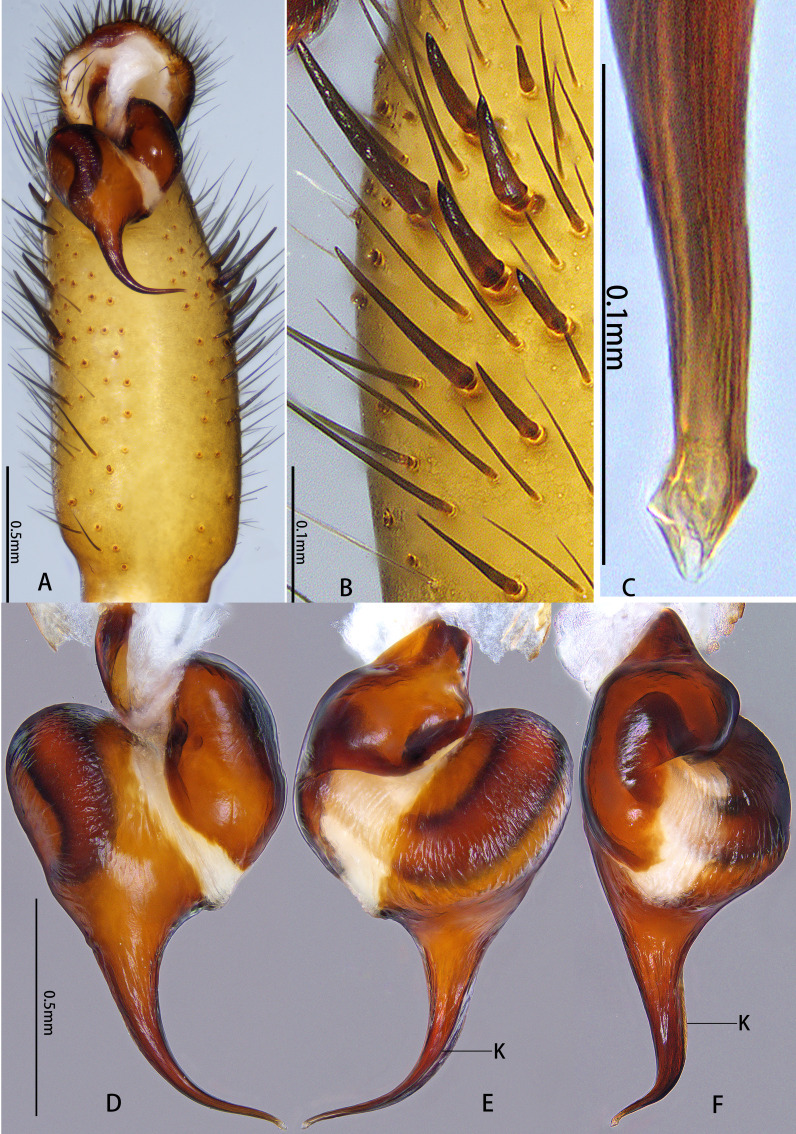
Palp of *Latouchiawenchuan* sp. nov., holotype male. **A** Palp; **B** Spines on the tibia of palp; **C** Apex of embolus; **D–F** Bulb. In prolateral (**C**), retrolateral (**B**, **F**), dorsal (**E**) and ventral (**A**, **D**) view. Abbreviations: K–keel.

**Figure 11.**
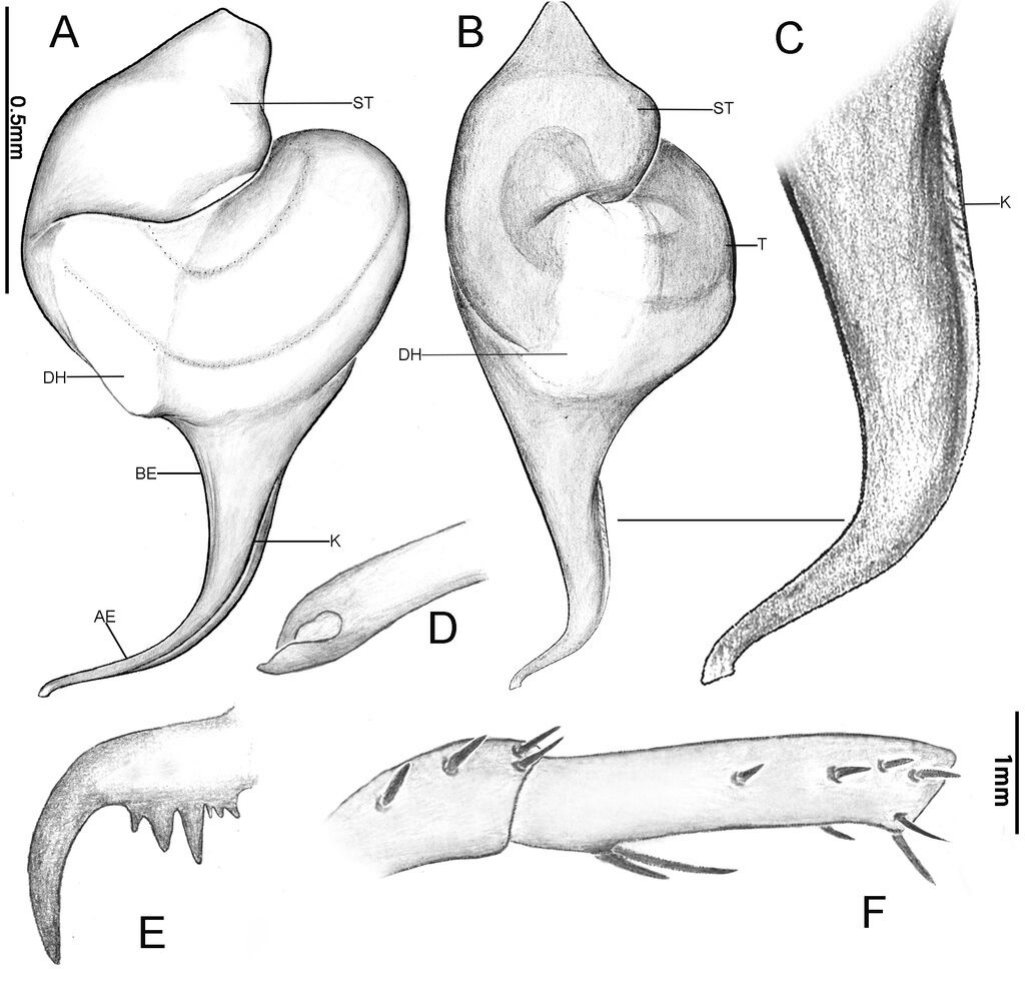
*Latouchiawenchuan* sp. nov., holotype male. **A, B** Palpal bulb; **C** Embolus; **D** Apex of embolus; **E** Claw of left leg I; **F** Patella and tibia of left leg II, showing the spination. In prolateral (**F**), retrolateral (**B**, **C, E**) and dorsal (**A**, **D**) view. Abbreviations: AE, apex of embolus; BE, base of embolus; DH, distal haematodocha; K–keel; ST–subtegulum; T–tegulum.

Palp 6.62 long (2.81, 0.99, 2.05, 0.77). Trichobothria on palpal tibia unmodified, present on proximal half part, on cymbium divided into unmodified and clavate forms, with latter occupying majority of trichobothrial area, while former sparsely distributed at distal end of trichobothrial area; count of trichobothria: tibia 1/3pd, 2/3rd, cymbium 2/4 unmodified and 1/3 clavate. Tibia cylindrical, slightly outwards in the middle ventral, with one lyriform organ on ventro-prolateral side of sub-distal part (Fig. 9E–F). Spination of tibia, Dmp (1-1-1-1)/(1-1-1-1), Mr (7)/(7). Palpal organ: tegulum oval; embolus approximately 1.3 times the tegulum width, with one elevated embolic keel extending from retrolateral side of proximal one-third part of embolus to distal one-third part of ventral side (Fig. 10D–F); embolus slight bent to retrolateral at distal one-third part of embolus; apex of embolus with an apophysis (Fig. 10C).

#### Diagnosis

The male of new species morphologically resembles those of geographically close *L.yaoi* sp. nov. on the general morphology of palpal bulb and the pattern of spines on the palpal tibia, but it can be distinguished from *L.yaoi* by the relatively low keel of embolus and the reduction of number of spines on male palpal tibia (Fig. 9E–F and Fig. 13F–G). Another species known only from a historical female specimen from the same wider zone is *L.davidi* (i.e. from Moupingzhen, Sichuan). Whilst direct comparisons are problematic due to sexual dimorphism, the male *L.wenchuan* sp. nov. lacks stridulatory ridges on the retrolateral checlicerae as found in the female of *L.davidi* (Decae and Caranhac 2020), but which may be expected in the undescribed male as similar structures are found in both sexes on *L.stridulans* (Decae et al. 2019).

#### Etymology

The specific epithet is derived from the type locality; noun in apposition.

#### Distribution

Known only from the type locality of Sichuan, China (Fig. 17).

#### DNA barcode

AAAGATATTGGAACTTTATATATGATTTTTGGTGTATGATCGGCTATGCTAGGGACTGCTATAAGAGTAATTATTCGAGTTGAGCTTGGTCAGGTTGGTAGATTGTTTGGGGATGATCATTTGTATAATGTTATTGTTACTGCTCATGCTTTAGTTATGATTTTTTTTATAGTTATGCCTATTATAATTGGTGGTTTTGGGAATTGATTGCTTCCATTAATGATTGGTTCACCAGATATGGCTTTTCCTCGTATAAATAATTTAAGATTTTGATTGCTTTTCCCTTCTTTATTTTTATTGTTGTTATCTTCTATAACGGATATTGGGGTGGGTGCTGGATGGACTATTTATCCTCCATTATCTTCTGATTTAGGACATAGAGGAGGAGGGGTAGATTTTGCTATTTTTTCTCTTCATTTGGCTGGAGGGTCTTCAGTAATAGGTTCTATTAATTTTATTTCTACTATTTTTAATATACGTCCTTTTGGGATAACAATAGAACGAGTTCCTTTATTTGTGTGATCTGTGTTAATTACAACTATTTTGCTTTTATTGTCTTTGCCAGTTTTAGCTGGAGCTATTACTATACTATTAACTGATCGAAATTTTAATACTTCATTTTTTGATCCTGCTGGTGGTGGGGATCCGGTATTATTTCAGCATTTGTTTTGATTTTTTGGTCAC (GenBank accession number: PQ585637).

### Latouchia yaoi

Hao, Yu & Zhang sp. nov.

51128E69-0B84-58EA-87D5-1AF16401264F

5E6EE817-2BF0-4943-89CB-5D7AA980ED8B

#### Materials

- **Type status:** Holotype. **Occurrence:** recordedBy: K. Yu & Y. Liang; sex: male (raised by L. Hao and matured in September 2023); occurrenceID: 9F849FFB-0671-5D0F-B87E-2D3C9385C159; **Taxon:** scientificName: *Latouchiayaoi* sp. nov.; **Location:** country: China; stateProvince: Shaanxi; county: Hanzhong; locality: Wuxiang Town, near Tiantai National Forest Park; verbatimElevation: 859 m; verbatimCoordinates: 33.2450°N, 107.0528°E; **Identification:** identifiedBy: L. Hao; **Event:** eventDate: 28 January 2023; **Record Level:** institutionCode: MHBU-ARA-10000056; KYUARA#1984
- **Type status:** Paratype. **Occurrence:** recordedBy: K. Yu & Y. Liang; sex: female; occurrenceID: 293B63F8-DB8B-510B-AACE-DF988169EB45; **Taxon:** scientificName: *Latouchiayaoi* sp. nov.; **Location:** country: China; stateProvince: Shaanxi; county: Hanzhong; locality: Wuxiang Town, near Tiantai National Forest Park; verbatimElevation: 859 m; verbatimCoordinates: 33.2450°N, 107.0528°E; **Identification:** identifiedBy: L. Hao; **Event:** eventDate: 7 February 2023; **Record Level:** institutionCode: MHBU-ARA-10000057; KYUARA#0059
- **Type status:** Paratype. **Occurrence:** recordedBy: K. Yu & Y. Liang; sex: female; occurrenceID: D467B434-D070-576A-B5C7-626D290DE468; **Taxon:** scientificName: *Latouchiayaoi* sp. nov.; **Location:** country: China; stateProvince: Shaanxi; county: Hanzhong; locality: Wuxiang Town, near Tiantai National Forest Park; verbatimElevation: 859 m; verbatimCoordinates: 33.2450°N, 107.0528°E; **Identification:** identifiedBy: L. Hao; **Event:** eventDate: 7 February 2023; **Record Level:** institutionCode: MHBU-ARA-10000058; KYUARA#0060
- **Type status:** Paratype. **Occurrence:** recordedBy: K. Yu & Y. Yang; sex: female; occurrenceID: B46D3ADD-607F-58D8-A457-0888958F1B16; **Taxon:** scientificName: *Latouchiayaoi* sp. nov.; **Location:** country: China; stateProvince: Shaanxi; county: Hanzhong; locality: Shengshui Town, Majiazui Village, near Shengshui Temple; verbatimElevation: 540 m; verbatimCoordinates: 33.0351°N, 107.1120°E; **Identification:** identifiedBy: L. Hao; **Event:** eventDate: 28 April 2020; **Record Level:** institutionCode: MHBU-ARA-10000059; KYUARA#1986

#### Description

**Figure 12.**
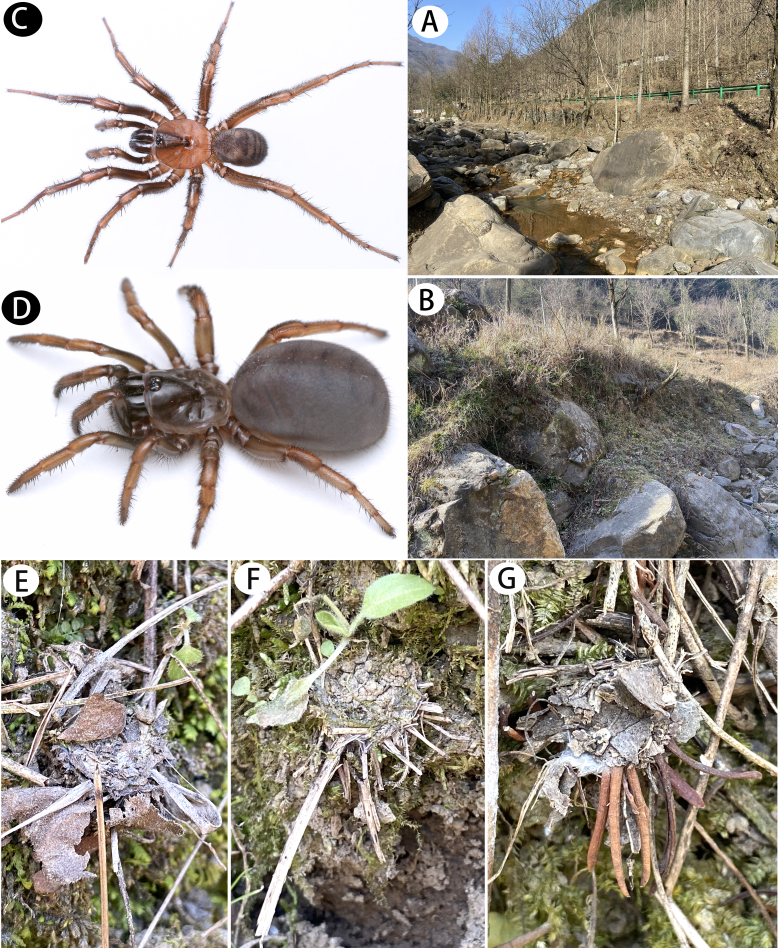
*Latouchiayaoi* sp. nov. **A, B** Habitats in the type locality; **C** Living holotype male; **D** Living paratype female; **E–G** Entrances of burrow, trapdoor closed. Photos: Kun Yu.

**Figure 13.**
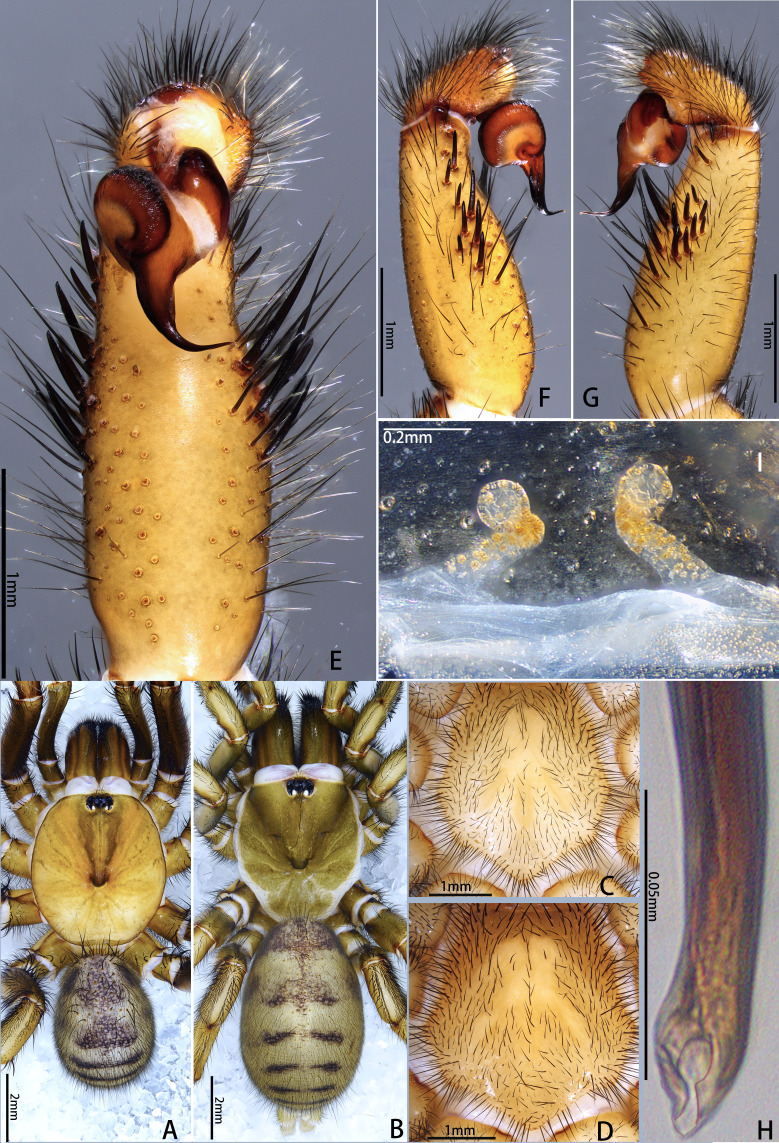
*Latouchiayaoi* sp. nov. **A**, **C**, **E–H** Holotype male; **B**, **D**, **I** Paratype female. **A, B** Habitus; **C, D** Sternums; **E–G** Palp; **H** Apex of embolus; **I** Vulva. In prolateral (**F**, **H**), retrolateral (**G**), dorsal (**A**, **B**, **I**) and ventral (**C–E**) view.

**Male** (Holotype, MHBU-ARA-10000056). Colouration in ethanol (Fig. 13A; for colouration of living holotype male, see Fig. 12C). Carapace yellowish-brown, chelicerae darker than carapace, with eye mound, fovea and outer edge of carapace darker; area between eye mound and fovea with two slightly darker longitudinal colour bands. Legs yellowish-brown, with femora gradually transitioning to slightly deeper hue from proximal to distal, darker than rest of legs. Opisthosoma: dorsal side grey, with distinct dark pattern; ventral side with dense black fur, slightly darker than dorsal side; booklung covers yellow. Ventral side of whole body generally brighter than dorsal side; sigilla slightly darker than rest of sternum (Fig. 13C).

Total length 10.07. Carapace 5.21 long, 4.60 wide; opisthosoma 4.84 long, 3.59 wide. Eye group 0.47 long, 0.53 wide anteriorly, 0.65 wide posteriorly; MOA 0.35 long, front width 0.23, back width 0.45. Eye diameters and interdistance: AME 0.14, ALE 0.16, PME 0.12, PLE 0.17, AME–AME 0.12, AME–ALE 0.11, ALE–PLE 0.15, PME– PME 0.35, PME–PLE 0.03. Palpal coxa 1.78 long, 1.01 wide, bearing 11/14 spinules on prolateral-proximal corner. Sternum 3.04 long, 2.63 wide. Labium 0.43 long, 0.84 wide, without cuspule or spinule. Chelicerae without stridulatory ridges; rastellum of left and right chelicerae carrying six and five stout spines, respectively; chelicerae groove with 5/5 and 3/3 teeth of different sizes on promargin and retromargin, respectively.

Leg formula 4123; measurements: Ⅰ 13.59 (3.35, 1.49, 3.68, 3.12, 1.95), II 12.78 (3.40, 1.24, 3.33, 3.10, 1.71), III 11.80 (2.81, 1.06, 2.48, 3.13, 2.32), IV 17.96 (5.47, 2.26, 4.30, 3.91, 2.02). Spines on femora to metatarsi of legs I–II straight, sword-like (typical); spines on the prolateral patellae of leg I-II typical, ventrally strong on leg I (especially distally), tip hooked, but absent on leg II; spines on prolateral tibiae of legs I few, absent on leg III, except apically, ventrally more elongate and expansive on both legs, two especially elongate adjacent spines proximally on leg II (Fig. 15D and Fig. 16c). Spination of leg I, patellae, Drv (3)/(1-3), Mpd (1-1-2)/(1-1-2), Mpv (1-1-1)/(1-1-2); tibiae, Mp (1-1-2-1-1)/(1-1-1-1-2), rv (2-2-2-1-3)/(1-2-2-2-3); metatarsi, Dpv (2)/(1), rv (1-1-1-1)/(1-1-1-1). Spination of leg II, patellae, Dpd (1-2)/(1-3), Drd (2)/(2); tibiae, Dp (2)/(1), Pv (3)/(3), Dv (1)/(1); metatarsi, Mrv (1)/(1-1). Tibia III unmodified. Trichobothria of legs present on proximal one-third part of tibiae I–II, half of tibiae III, proximal one-fourth part of tibia IV, distal half of metatarsi I–III, distal two-thirds part of metatarsus IV and dorsal side of all tarsi; trichobothria on tibiae I–IV and metatarsi I–IV unmodified; trichobothria on tarsi I–III divided into unmodified and clavate forms, with former irregularly distributed across almost entire dorsal surface and latter only present in proximal half, clavate form of trichobothria not observerd on tarsi IV. Count of trichobothria on legs: I, tibia 5/3pd and 4/5rd, metatarsus 8/8, tarsus 10/10 unmodified and 4/4 clavate; II, tibia 5/5pd and 5/5rd, metatarsus 9/9, tarsus 3/10 unmodified and ?/4 clavate; III, tibia 4/4pd and 5/3rd, metatarsus 8/8, tarsus 18/15 unmodified and 3/2 clavate; IV, tibia 5/6pd and 3/6rd, metatarsus 5/5, tarsus 12/11 unmodified. Tarsal claws: paired claws with 8–10 teeth (Fig. 15F); unpaired claw bare, without denticle.

**Figure 14.**
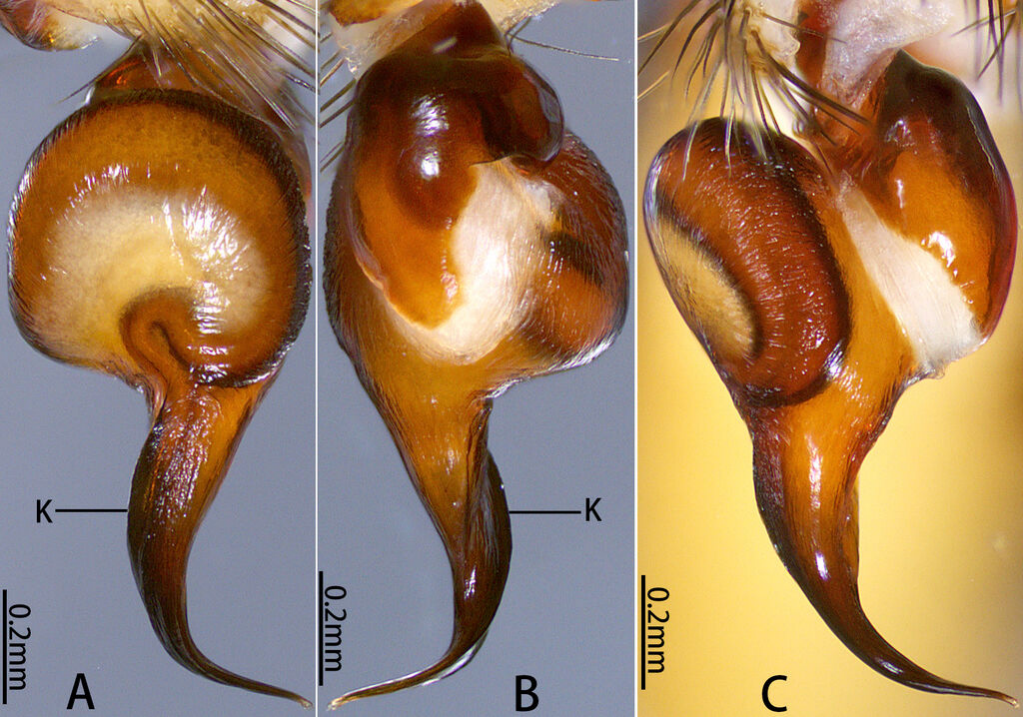
Palpal bulb of *Latouchiayaoi* sp. nov., holotype male. In prolateral (**A**), retrolateral (**B**) and ventral (**C**) view. Abbreviations: K–keel.

Palp 6.35 long (2.57, 1.08, 2.11, 0.59). Trichobothria on palpal tibia unmodified, present on proximal one-third part, on cymbium divided into unmodified and clavate forms, with latter occupying majority of trichobothrial area, while former sparsely distributed at distal end of trichobothrial area; count of trichobothria: tibia 4/4pd, 4/2rd cymbium, 4/3 unmodified and 5/5 clavate. Tibia cylindrical, transitioning to slightly narrow from proximal to distal, the distal end is half the thickness of the proximal end, with one lyriform organ on ventro-prolateral side of sub-distal part. Short strong spines of palp tibia, Mp (1-2-1-2-2-1-1)/(1-2-1-1-2-1), Mr (2-3-2-1-1)/(1-1-3-2-2-1), Drv (2)/(2). Palpal organ: tegulum oval (Fig. 14A–C); embolus base thick, the length of embolus approximately 1.5 times the width of tegulum, with one distally elevated embolic keel extending from retrolateral side of sub-basal part of embolus to dorsal side of sub-distal part; embolic keel smooth; apex of embolus slender, gradually tapering to sharp tip (Fig. 13H).

**Female** (Paratype, MHBU-ARA-10000057). Colouration in ethanol (Fig. 13B; for colouration of living paratype fermale, see Fig. 12D). Carapace like male, but slightly darker, eye mound and fovea darker; chelicerae yellowish-brown similar to carapace. Colour between palp and each leg without significant difference, overall similar in colour to carapace. Opisthosoma: dorsal side colour like male, but slightly lighter, with distinct dark pattern. Ventral side of whole body generally brighter than dorsal side; booklung covers paler than remaining ventral side. Palpal coxa and labium slightly darker, sigilla lighter than rest of the sternum (Fig. 13D).

Total length 14.17. Carapace 5.99 long, 4.65 wide; opisthosoma 8.17 long, 5.46 wide. Eye group 0.62 long, 0.46 wide anteriorly, 0.78 wide posteriorly; MOA 0.46 long, front width 0.27, back width 0.47. Eye diameters and interdistance: AME 0.13, ALE 0.29, PME 0.18, PLE 0.28, AME–AME 0.18, AME–ALE 0.17, ALE–PLE 0.16, PME–PME 0.32, PME–PLE 0.10. Palpal coxa 2.05 long, 1.23 wide, bearing 15/18 cuspules on prolateral-proximal corner. Sternum 3.62 long, 3.31 wide. Labium 0.97 long, 1.27 wide, without spinules or cuspule. Chelicerae without stridulatory ridges; rastellum of left and right chelicerae carrying six and seven stout spines, respectively; chelicerae groove with 5/5 and 3/3 teeth of different sizes on promargin and retromargin, respectively.

**Figure 15.**
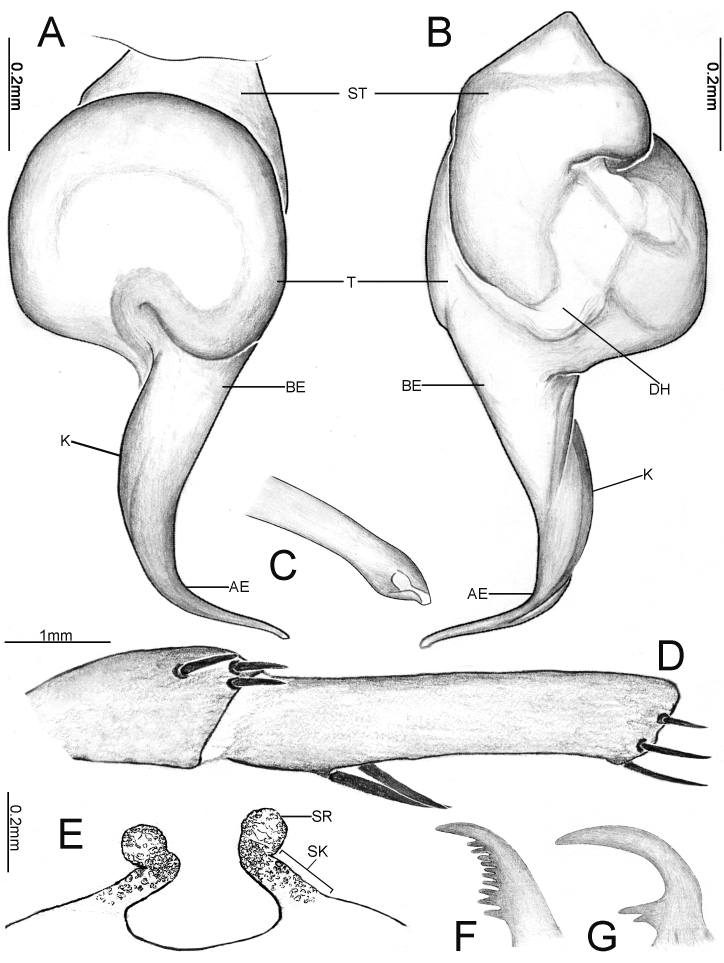
*Latouchiayaoi* sp. nov. **A–D**, **F** Male; **E**, **G** Female. **A, B** Palpal bulb; **C** Apex of embolus; **D** Patella and tibia of left leg II, showing the spination; **E** Vulva; **F, G** Paired claws of left leg I. In prolateral (**A**, **C**, **D**), retrolateral (**B**, **F**, **G**) and dorsal (**E**) view. Abbreviations: DH, distal haematodocha; E–embolus; K–keel; SR–sperm receptacle; ST–subtegulum; SK–stalk; T–tegulum.

Leg formula 4123; measurements: Ⅰ 11.27 (3.92, 2.10, 2.61, 1.50, 1.14), II 9.53 (3.38, 1.73, 1.65, 1.58, 1.19), III 7.93 (2.64, 1.48, 1.17, 1.41, 1.23), IV 13.24 (4.25, 1.94, 2.61, 2.65, 1.79). Spines of legs I–II primarily distributed on p, pd, r and rv of tibia, as well as p, pv, r and rv of metatarsus and tarsus; the tip of most spines weakly curved downwards, forming slight hook-shape; some spines on rv longer and not curved at tip. Leg III strong; groups of short strong spines on apical and prodorsal sides of patella; tibia III shortened, without demi-saddle shape; metatarsus with numerous short, strong spines closely grouped in dorsal area, slender spines on ventro-distal. Groups of short strong spines on prolateral patella and slender spines on ventral metatarsus of Leg IV. Tarsus III–IV with ventro-distal group of stiff bristles and few slender spines. Trichobothria of legs present on proximal one-third part of tibiae I–III, proximal half part of tibia IV, distal half of metatarsi I–IV and proximal two-thirds of all tarsi; trichobothria on tibiae I–IV and metatarsi I–IV unmodified; trichobothria on tarsi I–III divided into unmodified and clavate forms, with latter only present in proximal half of tarsus, trichobothria on tarsi IV without clavate forms. Count of trichobothria on legs: I, tibia 4/4pd and 5/3rd, metatarsus 8/6, tarsus 13/11 unmodified and 5/4 clavate; II, tibia 4/5pd and 4/4rd, metatarsus 9/7, tarsus 8/7 unmodified and 4/4 clavate; III, tibia 4/4pd and 5/4rd, metatarsus 5/5, tarsus 12/9 unmodified and 3/3 clavate; IV, tibia 7/6pd and 6/6d, metatarsus 7/6, tarsus 12/14 unmodified. Tarsal claws: all paired claws with two teeth (Fig. 15G); unpaired claw bare, without denticle. Palp 10.04 long (3.76, 1.44, 2.28, 2.56), spines on palp distributed on p, pv, r and rv of tibia and tarsus; palpal tarsal claw with one basal tooth.

Vulva (Fig. 13I and Fig. 15E). Two separate sperm receptacles connected to atrium via slender stalk, stalk inwardly inclined, sperm receptacles nearly spherical, tilted outwards at approximately 45°. Tan glandular pores present on distal two-thirds of stalk and entire sperm receptacle. Glandular pores also present on basal one-third of stalk, atrium, as well as uterus externus.

#### Diagnosis

The male similar to *Latouchiawenchuan* sp. nov. For the differences, see *Latouchiawenchuan* sp. nov.. The male can be easily distinguished from the geographically close *L.jinyun* sp. nov. by whether spines are present on the palpal tibia.

#### Etymology

The specific epithet is in honour to Mr. Yao Yang, a good friend of the second author who, together with the second author, collected the first specimen of this new species.

#### Distribution

**Figure 17.**
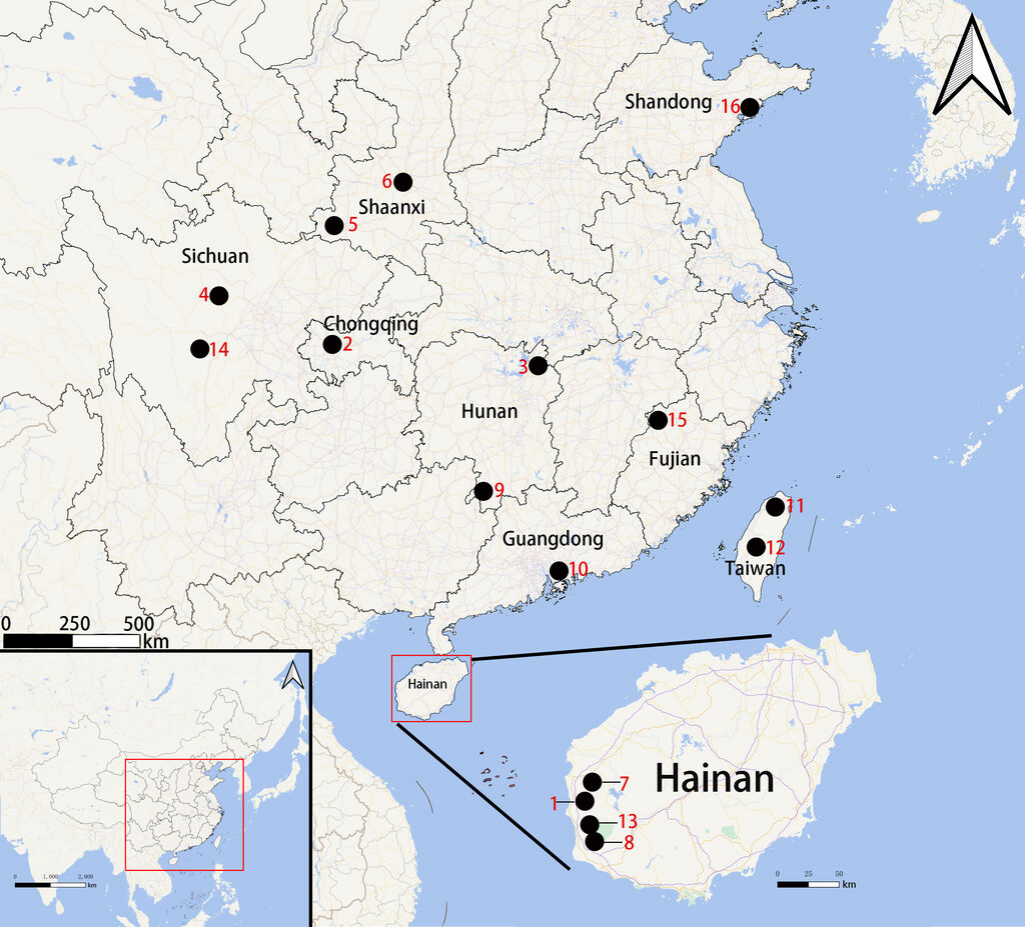
The type localities of *Latouchia* species of China: *L.calcicola* sp. nov. (1), *L.jinyun* sp. nov. (2), *L.linmufu* sp. nov. (3), *L.wenchuan* sp. nov. (4), *L.yaoi* sp. nov. (5), *L.cornuta* (6), *L.wenruni* (7), *L.yuanjingae* (8), *L.hunanensis* (9), *L.rufa* (10), *L.formosensis* (11), *L.formosensissmithi* (12), *L.yejiei* (13), *L.davidi* (14), *L.fossoria* (15) and *L.pavlovi* (16). The type locality of *L.vinhiensis* is uncertain.

Known only from the type locality of Shaanxi, China (Fig. 17).

#### Biology

The burrows of this species were found on slopes along roadsides or small riverbanks (Fig. 12A and B). The rim of the burrow entrance is sometimes adorned with dried plant materials (Fig. 12 E–G). The holotype male was matured in September in captivity; some females from Wuxiang Town were observed carrying spiderlings (likely to be third instar) in late January, even though the temperature at the time was only between minus one and seven degrees Celsius. Some individuals have been observed exhibiting self-burying behaviour in captivity, which involves filling the burrow with soil, leaving just enough space at the bottom of the burrow to accommodate their bodies and living within this confined space. This behaviour typically occurs after a substantial meal, although the purpose remains unclear; the longest observed period for a female in this state was over 40 days.

#### DNA barcode

AAAGATATTGGAACTTTGTATATAATTTTTGGGGTGTGGTCGGCTATGGTAGGGACTGCAATAAGAGTAATCATTCGAATTGAGCTTGGACAAGTTGGAAGATTATTTGGTGATGATCATTTATATAATGTTGTTGTTACTGCTCATGCTTTAGTTATAATTTTTTTTATAGTAATACCTATTATAATTGGGGGCTTTGGAAATTGACTGCTTCCAATAATAATTGGTTGTCCAGATATAGCTTTTCCACGAATGAATAATTTAAGATTTTGATTGCTTCCTCCTTCTCTGTTTTTGCTTTTGTTGTCTTCTATAACAGATGTCGGAGTGGGTGCCGGTTGAACTATTTATCCTCCTTTGTCTTCTGAACTTGGCCATAGAGGTGGAGGGATAGATTTTGCTATTTTTTCTCTTCATTTGGCTGGTGGGTCTTCGGTGATGGGTTCTATTAATTTTATTTCTACAATTTTAAATATGCGTCCTTTTGGAATGATAATGGAGCGAGTTCCTTTATTCGTGTGATCTGTATTAATTACTACTATTTTATTGTTATTATCTTTACCTGTATTGGCTGGGGCTATTACCATATTATTAACTGATCGAAATTTTAATACTTCGTTTTTTGATCCTGCTGGTGGGGGGGATCCTGTGTTGTTTCAGCATTTGTTTTGATTTTTTGGTCA (GenBank accession number: PQ585636).

## Supplementary Material

XML Treatment for Latouchia

XML Treatment for Latouchia calcicola

XML Treatment for Latouchia fossoria

XML Treatment for Latouchia jinyun

XML Treatment for Latouchia linmufu

XML Treatment for Latouchia wenchuan

XML Treatment for Latouchia yaoi

## Figures and Tables

**Figure 4a.**
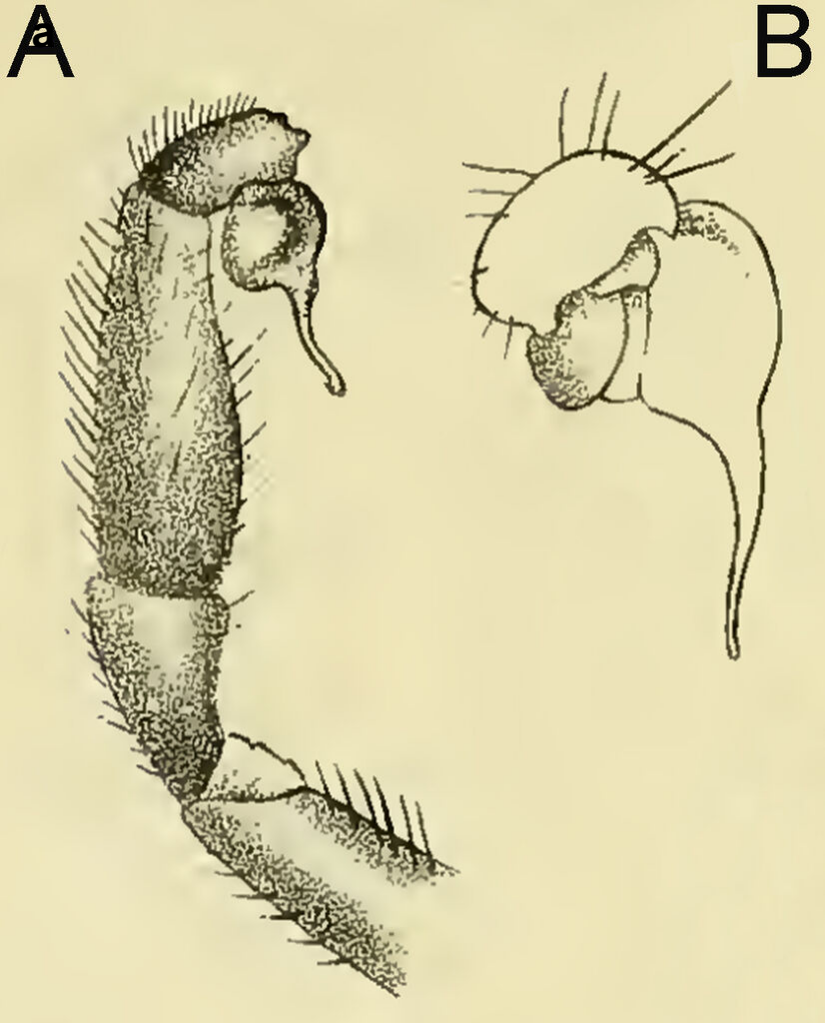
Original illustrations of *L.swinhoei* , modified from Pocock 1901, Figs. 3 and 3a.

**Figure 4b.**
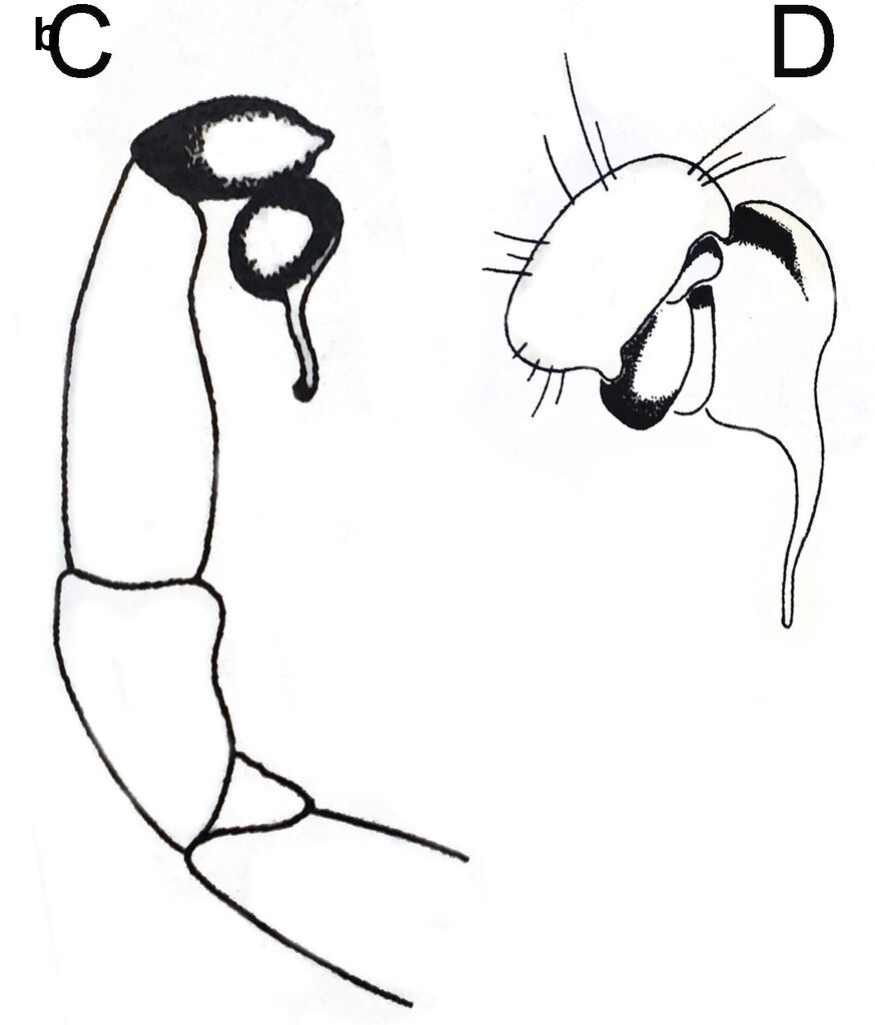
Later illustrations of alleged male "*L.fossoria* Pocock 1901", modified from Song et al. (1999), Figs. 16Q–R.

**Figure 16a.**
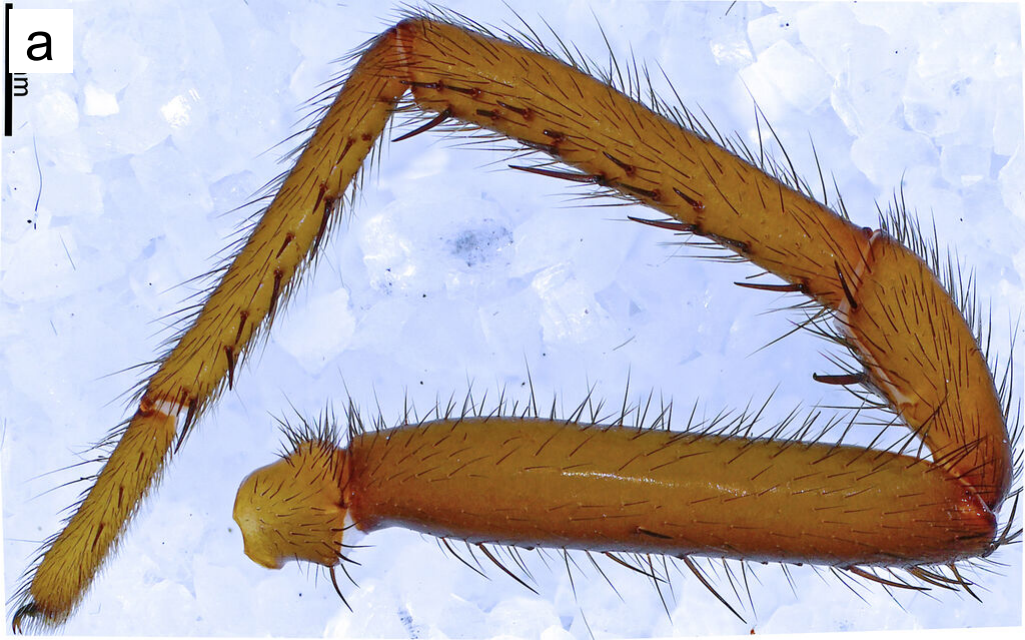
*Latouchiawenchuan* sp. nov. Right leg I;

**Figure 16b.**
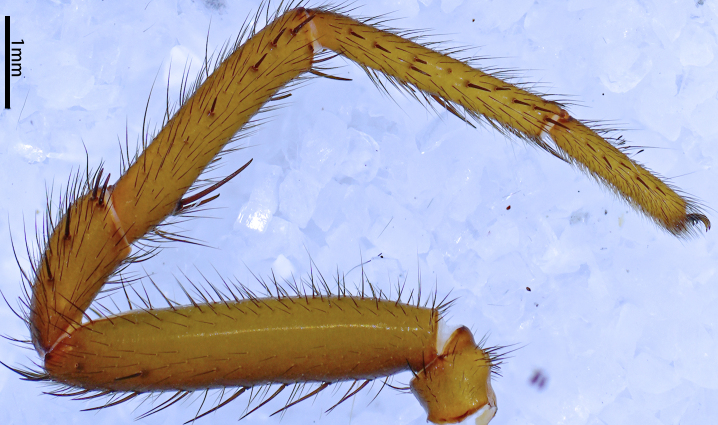
*Latouchiawenchuan* sp. nov. Left leg II;

**Figure 16c.**
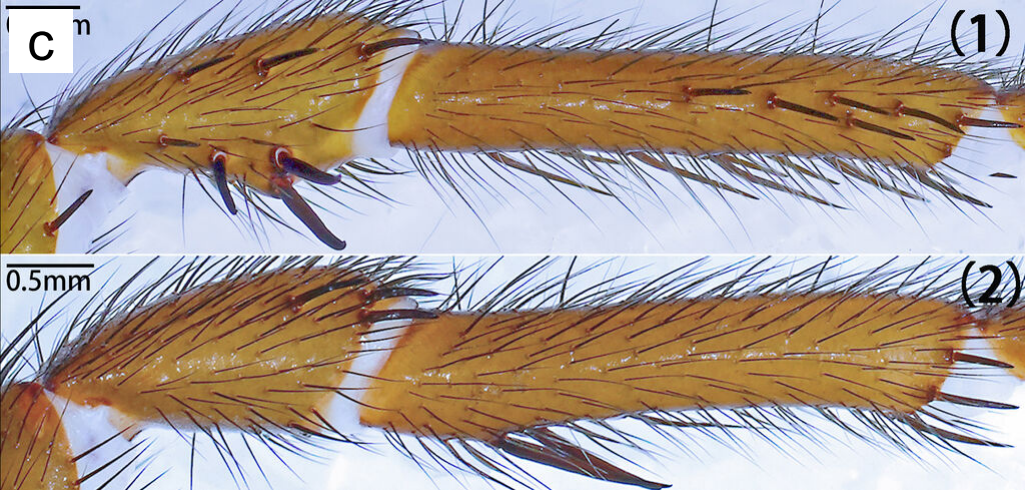
*Latouchiayaoi* sp. nov. (1) left leg I, (2) left leg II;

**Figure 16d.**
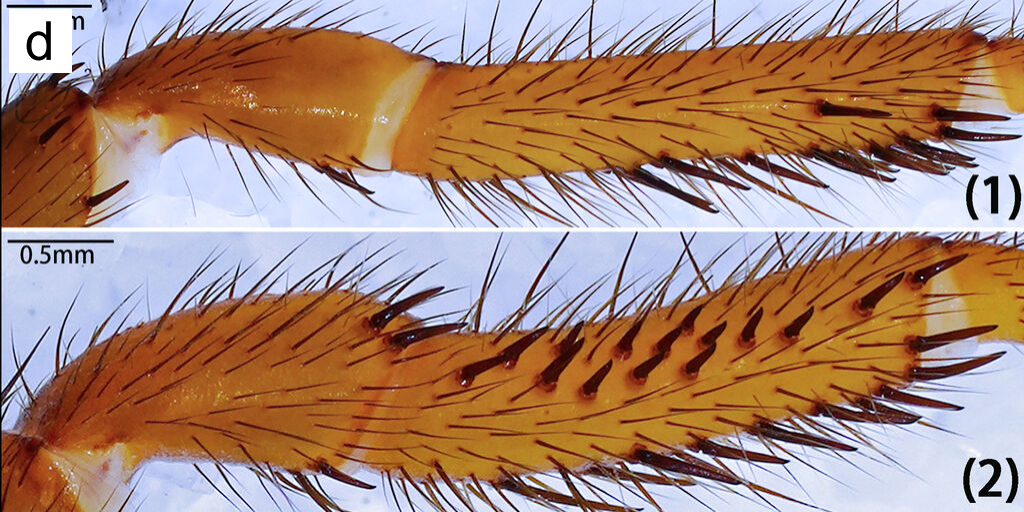
*Latouchiajinyun* sp. nov. (1) left leg I, (2) left leg II;

**Figure 16e.**
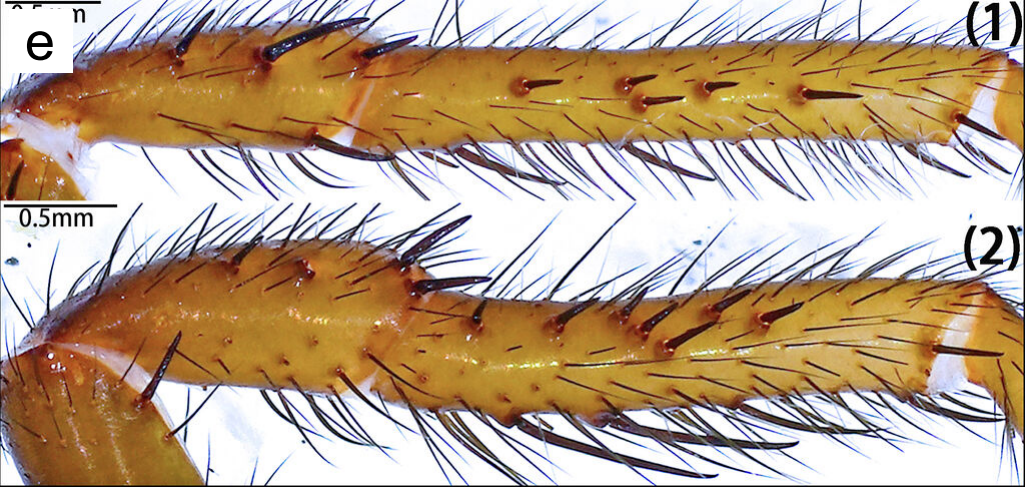
*Latouchialinmufu* sp. nov. (1) left leg I, (2) left leg II;

**Figure 16f.**
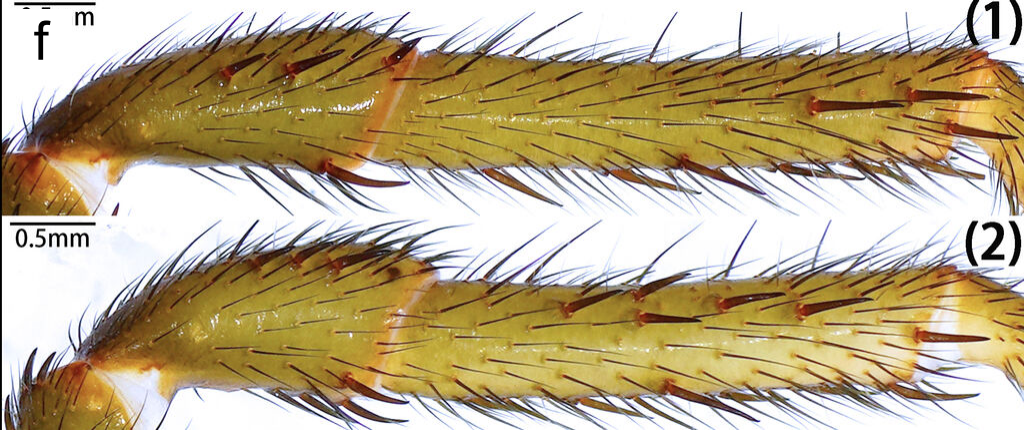
*Latouchiacalcicola* sp. nov. (1) left leg I, (2) left leg II.
